# Phylogenomics, plastome structure and species identification in *Mahonia* (Berberidaceae)

**DOI:** 10.1186/s12864-022-08964-0

**Published:** 2022-11-23

**Authors:** Ruchang Tong, Chaoxia Gui, Yu Zhang, Na Su, Xiaoqi Hou, Meng Liu, Zhaoping Yang, Bing Kang, Zhaoyang Chang, Florian Jabbour, Liang Zhao

**Affiliations:** 1 College of Life Sciences, Northwest A&F University, Yangling, 712100 Shaanxi China; 2 Herbarium of Northwest A&F University, Yangling, 712100 Shaanxi China; 3 Guiyang Botanical Garden, Guiyang, 550009 Guizhou China; 4 Xishuangbanna Tropical Botanical Garden, Chinese Academy of Sciences, Menglun, Mengla, 666303 Yunnan China; 5 College of Life Sciences, Tarim University, Alar, 843300 Xinjiang China; 6 Institut de Systématique, Evolution, Biodiversité (ISYEB), Muséum National d’Histoire Naturelle, CNRS, Sorbonne Université, EPHE, Université Des Antilles, 75005 Paris, France

**Keywords:** Berberidaceae, Leaf epidermal characters, *Mahonia*, Plastome, Special barcodes

## Abstract

**Background:**

Elucidating the phylogenetic relationships within species-rich genera is essential but challenging, especially when lineages are assumed to have been going through radiation events. *Mahonia* Nutt. (Berberidaceae) is a genus with cosmopolitan distribution, comprising approximately 100 species, two of which are known as Caulis Mahoniae (*M*. *bealei* and *M*. *fortunei*) with crucial pharmacological significance in Chinese herbal medicine. *Mahonia* is a taxonomically challenging genus, and intrageneric phylogenetic relationships still need to be explored using genome data. Universal DNA barcodes and floral morphological attributes have limited discriminatory power in *Mahonia*.

**Results:**

We sequenced 17 representative plastomes and integrated three published plastome data together to conduct comparative and phylogenetic analyses. We found that *Mahonia* and *Berberis* share a large IR expansion (~ 12 kb), which is recognized as a typical character of Berberideae. Repeated sequences are revealed in the species of *Mahonia*, which are valuable for further population genetic studies. Using a comparative plastome analysis, we determined eight hypervariable regions whose discriminative power is comparable to that of the whole plastid genomes. The incongruence of the ITS and the plastome tree topologies may be ascribed to ancestral hybridization events and/or to incomplete lineage sorting. In addition, we suggest that leaf epidermal characters could help to distinguish closely related species in *Mahonia*.

**Conclusions:**

We propose an integrative approach combining special barcodes and micromorphological traits to circumscribe *Mahonia* species. The results cast a new light on the development of an integrative method for accurate species circumscription and provide abundant genetic resources for further research on *Mahonia*.

**Supplementary Information:**

The online version contains supplementary material available at 10.1186/s12864-022-08964-0.

## Background

The Berberidaceae (Ranunculales) is an early-diverging eudicot plant family comprising 19 genera, including the newly proposed *Alloberberis* P.H. Raven ex C.C. Yu & K.F. Chung and *Moranothamnus* P.H. Raven ex C.C. Yu & K.F. Chung [1, 2]. The 680 + Berberidaceae species are predominantly distributed in northern temperate zones extending to Andean South America and northern Africa [3–5]. The barberry family is traditionally known for its morphological diversity, intercontinental discontinuous distribution and medicinal utilization [5, 6].

**Fig. 1:**
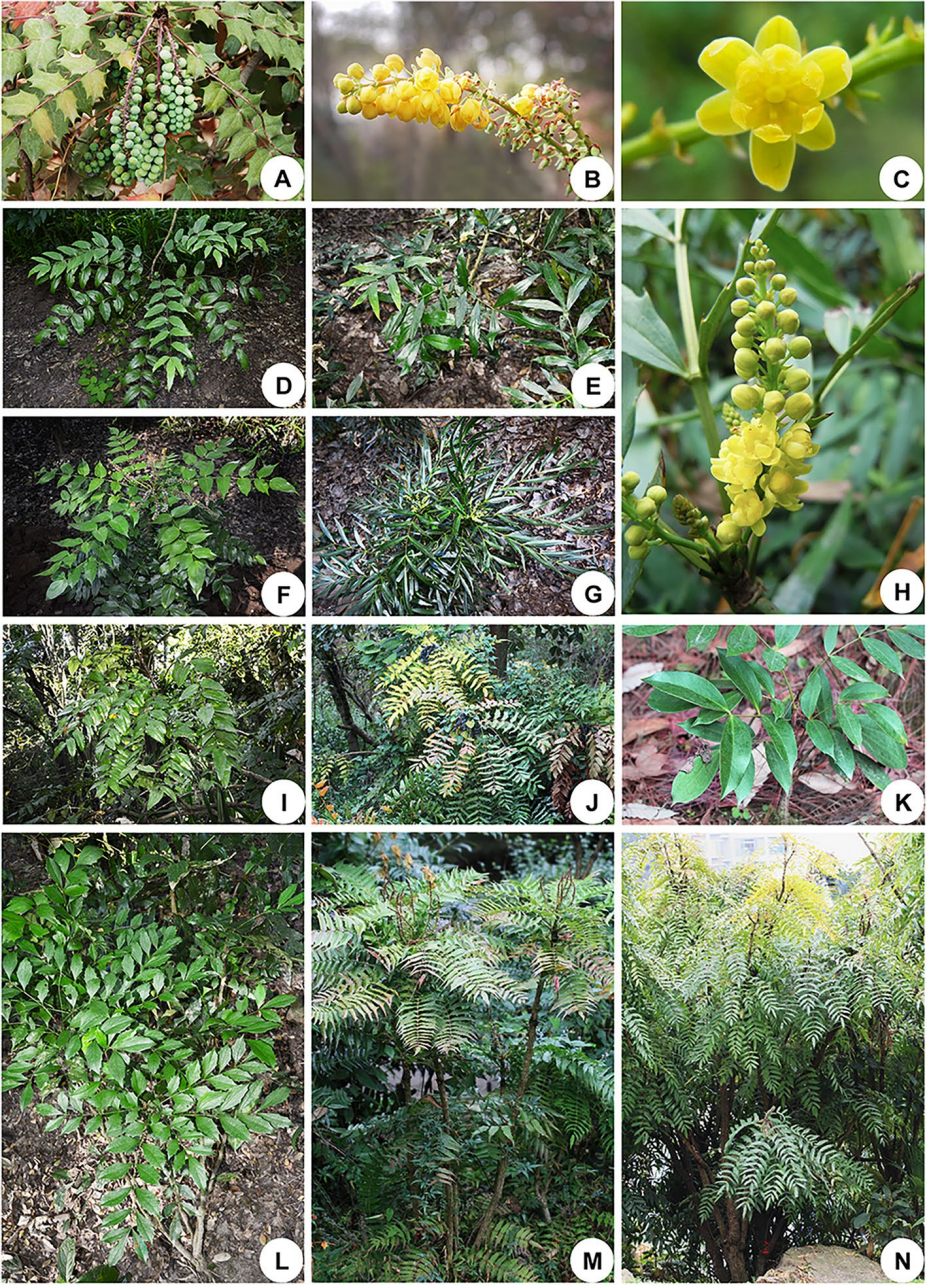
Morphological diversity of *Mahonia* species. **A B** *M*. *bealei*. **A** fruits. **B** racemose inflorescence. **D** *M. hancockiana*. **C E H** *M. fortunei*. **C** a single flower. **E** compound leaves. **H** racemose inflorescence. **F** *M. fordii*. **G** *M. eurybracteata* subsp. *ganpinensis*. **I** *M. napaulensis*. **J** *M. bordinieri*. **K** *M. shenii*. **L** *M. breviracema*. **M** *M. oiwakensis*. **N** *M. duclouxiana*

*Mahonia* Nutt. is the second largest genus in Berberidaceae, comprising about 100 species [7]. However, the precise number of *Mahonia* species remains ambiguous, as 33 species were synonymized in the Flora of China [4]. Morphologically, the species of *Mahonia* are easily distinguished from other angiosperm species by their evergreen odd-pinnately compound leaves, their leaflets margins with spinose dentation, and their spineless stems [4]. The species of *Mahonia* are distributed in East Asia and Western North America [1, 8], making the genus an emblematic example of a biogeographic disjunction. Besides, a few species of *Mahonia* are endemic to Europe, North Africa and South America [8]. Many species of *Mahonia* are broadly cultivated for horticulture (Fig. 1) and for their pharmacological properties [4, 7, 9]. For instance, the stems of *M*. *bealei* (Fortune) Carrière and *M*. *fortunei* (Lindl.) Fedde are known as Caulis Mahoniae with highly anti-inflammatory properties [10] and are included in the Chinese pharmacopoeia [9].

The position of the genus *Mahonia* remains intractable and has been discussed for a long time [1, 11, 12]. Traditionally, morphological and molecular evidence indicated that *Mahonia* was paraphyletic, with *Mahonia* sect. *Horridae* being sister to *Berberis* L. sensu stricto [5, 12, 13]. Although several authors held a view that *Mahonia* should be subsumed under a broadly defined *Berberis* (*Berberis *sensu lato) [12–14], a great majority of researchers advocate for a paraphyletic *Mahonia* because of its compound leaves that are distinct from the simple leaves of *Berberis* [7, 11, 12, 15]. Yu and Chung [1] proposed a new classification that divided *Berberis s.l.* into four monophyletic clades and establishing four new genera (*Berberis* ≡ *Berberis s.s.*, *Mahonia* ≡ core *Mahonia*, *Alloberberis* ≡ *Mahonia* sect. *Horridae*, and *Moranothamnus* ≡ *Berberis claireae*). This taxonomic treatment not only maintained the universally acceptable perception of *Berberis* but also resolved *Mahonia* as a monophyletic genus, which has been widely accepted by botanists in the fields of phylogenomics [16], taxonomy [17], and biogeography [8].

The genus *Mahonia* is taxonomically and phylogenetically challenging, owing to its considerable species richness, to the rapid diversification events that have punctuated its evolutionary history, and to the high similarity in the morphology of reproductive structures that hinders the easy and accurate identification of species [7, 8, 18]. Within *Mahonia*, floral organs are usually invariable in number and are arranged in whorls, and all *Mahonia* species bear similar yellow flowers and blue-black globose berries [4, 7].

Previously, a series of comparative plastome analyses found that a large IR expansion of over 12 kb occurred in *M*. *bealei*, which is unusual in plastome evolution [19, 20]. Using ITS (nuclear ribosomal DNA) and four DNA fragments (including the genes *accD*, *ndhF*, *rbcL* and the intergenic spacer *trnH*-*psbA*) of the plastid genome, Yu and Chung [1] proposed a new classification that recognized *Mahonia* as a distinctive monophyletic genus with strong support. The oldest reliable fossil records of *Mahonia* were collected in East Asia and used for biogeographic analyses [18]. On the basis of molecular dating estimates and comparison of leaf morphologies of extant *Mahonia* species, the researchers inferred that the genus *Mahonia* originated in Western North America and subsequently dispersed into East Asia. Notably, after the migration to East Asia, the genus *Mahonia* probably underwent a radiation event, leading to the current Eastern Asian biodiversity center [8, 18].

Phylogenetic analyses based on molecular datasets provide reasonable phylogenetic hypotheses [21–24]. However, it is disputable that using just a single line of evidence is sufficient to delimit species boundaries [25]. Therefore, multiple evidence (such as including plastome datasets, morphological traits, ecological traits) should be applied to modern systematics [26], in particular with respect to recently diverging lineages, such as *Mahonia*. Complete plastome data have proven to be effective in resolving phylogenetic relationships at a wide range of taxonomic levels [16]. The phylogenetic incongruence between the plastome tree and the nrDNA tree indicated that frequent hybridization has occurred between *Mahonia* and *Berberis* [2].

Characters of the apex of petals, the length of pedicels and bracts, the number of leaflets and spinose dentations, were used as critical morphological traits for discriminating the species of *Mahonia* [3, 4]. Given the stability and uniformity of micromorphological traits among taxa, researchers have undertaken a series of investigations to provide more evidence for the classification of *Mahonia* (e.g., floral anatomy [27]; seed micromorphology [28]; carpel micromorphology [29, 30]; sepal morphology [31]). Structural characters of leaf epidermis are usually constant and more accessible; they have been proven to possess great systematic significance in some complex taxa [32–34]. However, studies of leaf epidermal micromorphology with respect to the genus *Mahonia* is far from sufficient.

Here, we sequenced 17 representative complete plastomes of the genus *Mahonia*, and used 13 plastomes from GenBank to conduct comparative and phylogenetic analyses. Morphological and micromorphological traits of different species of *Mahonia* were recorded. We combined the evidence from the molecular and morphological data to resolve the phylogenetic relationships in *Mahonia*. Our goals are to 1) reconstruct phylogenetic relationships within *Mahonia* using nuclear internal transcribed spacer (ITS) and plastid genome sequences; 2) describe and interpret the plastome structure and evolution of *Mahonia*; 3) explore an integrative method for better distinguishing among *Mahonia* species.

## Results

### Plastome features of *Mahonia*

**Fig. 2:**
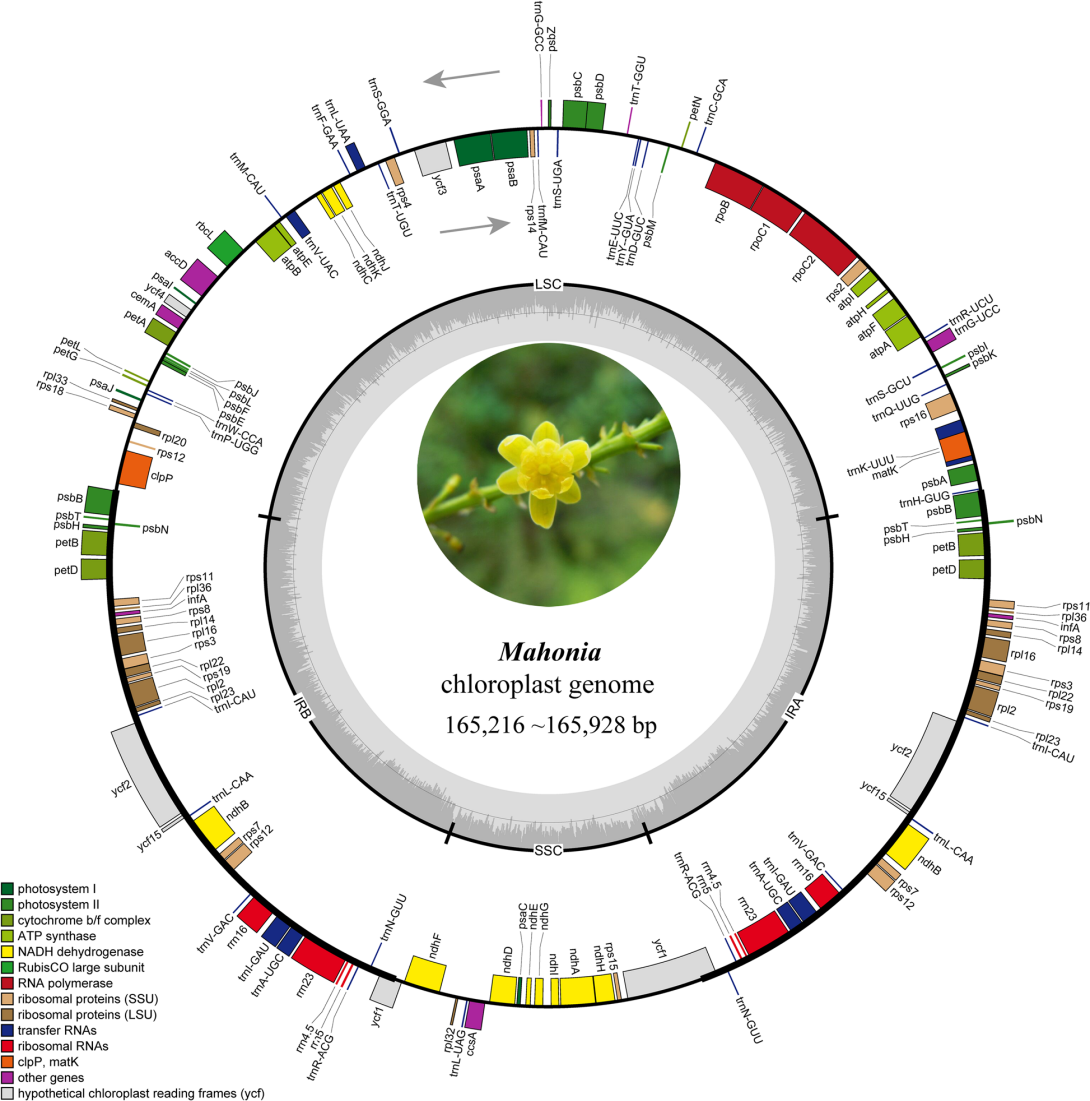
Gene map of *Mahonia* chloroplast genome. The two gray arrows indicate the direction of gene transcription. The dashed area in the inner circle indicates the GC content of the plastome. LSC: large-single-copy; SSC: small-single-copy; IR: inverted repeat

The number of raw paired-end reads for each plastome ranges from 15,790,898 (*M. breviracema* Y.S. Wang & P.G. Xiao RC611 [MZ158268]) to 25,347,900 (*M. duclouxiana* Gagnep. RC602 [MZ086770]) (Table 1). The assembled plastid genomes range from 165,216 bp (*M. napaulensis* DC. RC603 [MZ158275]) to 165,928 bp (*M. shenii* Chun RC609 [MZ158280]) in length with 38% to 38.1% genomic GC contents overall. The GC contents in inverted regions (IR, 41.1%–41.2%) are much higher than in the large single copy (LSC) and in the small single copy (SSC) regions (Table 1). The typical quadripartite configuration of these plastid genomes consisted of IR (36,641 bp–36,864 bp), which are separated by LSC (73,198 bp–73,703 bp) and SSC (18,563 bp–18,873 bp) regions (Fig. 2, Table 1).

**Table 1:** Summary of 17 complete chloroplast genomes of *Mahonia*

Species	Voucher number	Number of reads	Average depth of coverage ( ×)	Total size (bp)	Total GC%	LSC		SSC		IR	
						**size (bp)**	**GC%**	**size (bp)**	**GC%**	**size (bp)**	**GC%**
*M. bealei*	RC601	18,802,778	147.3	165,713	38%	73,516	36.4%	18,773	32.2%	36,712	41.2%
*M. duclouxiana*	RC602	25,347,900	1099.9	165,384	38.1%	73,477	36.4%	18,563	32.4%	36,672	41.2%
*M. napaulensis*	RC603	16,952,962	275.3	165,216	38.1%	73,313	36.5%	18,563	32.4%	36,670	41.2%
*M. cardiophylla*	RC604	18,211,456	537.9	165,559	38.1%	73,353	36.4%	18,802	32.3%	36,702	41.2%
*M. nitens*	RC605	19,474,194	1456.3	165,692	38.1%	73,703	36.4%	18,655	32.4%	36,667	41.2%
*M. gracilipes*	RC606	17,893,824	575.3	165,909	38%	73,451	36.4%	18,730	32.4%	36,864	41.1%
*M. polyodonta*	RC607	17,570,436	779.1	165,526	38.1%	73,280	36.5%	18,804	32.4%	36,721	41.2%
*M. bodinieri*	RC608	16,171,718	395.3	165,697	38%	73,594	36.4%	18,675	32.4%	36,714	41.1%
*M. shenii*	RC609	17,804,842	778.8	165,928	38.1%	73,528	36.4%	18,764	32.3%	36,818	41.1%
*M. oiwakensis*	RC610	22,462,532	80.4	165,267	38.1%	73,198	36.5%	18,721	32.4%	36,674	41.2%
*M. breviracema*	RC611	15,790,898	408.3	165,555	38.1%	73,365	36.4%	18,706	32.3%	36,742	41.1%
*M. fordii*	RC612	23,844,540	165.2	165,689	38%	73,558	36.4%	18,705	32.4%	36,713	41.1%
*M. hancockiana*	RC613	18,513,816	150.6	165,495	38.1%	73,483	36.4%	18,670	32.4%	36,671	41.2%
*M. eurybracteata*	RC614	15,910,170	389.3	165,612	38.1%	73,474	36.4%	18,734	32.4%	36,702	41.2%
*M. japonica*	RC615	24,350,910	310	165,711	38%	73,685	36.4%	18,676	32.4%	36,675	41.1%
*M. aquifolium*	RC616	19,912,290	37.4	165,689	38.1%	73,212	36.5%	18,873	32.4%	36,802	41.1%
*M. pinnata*	RC618	18,121,752	35.8	165,308	38.1%	73,313	36.5%	18,713	32.4%	36,641	41.2%

The *Mahonia* species we sequenced encode 113 unique genes, 34 of which are duplicated in the IR. A total of 79 protein-coding, 30 transfer RNA (tRNA) and four ribosomal RNA (rRNA) genes are successfully predicted. Each IR copy contains 23 protein-coding, seven tRNA and four rRNA genes. In total, 147 genes are included in the *Mahonia* plastid genomes we reconstructed (Table S1). There are 18 unique intron-containing genes in the plastid genomes. Sixteen genes (six tRNA and ten protein-coding genes) have a single intron, and the other two (*ycf3* and *clpP*) possess two introns.

### Comparative plastid genome analyses

**Fig. 3:**
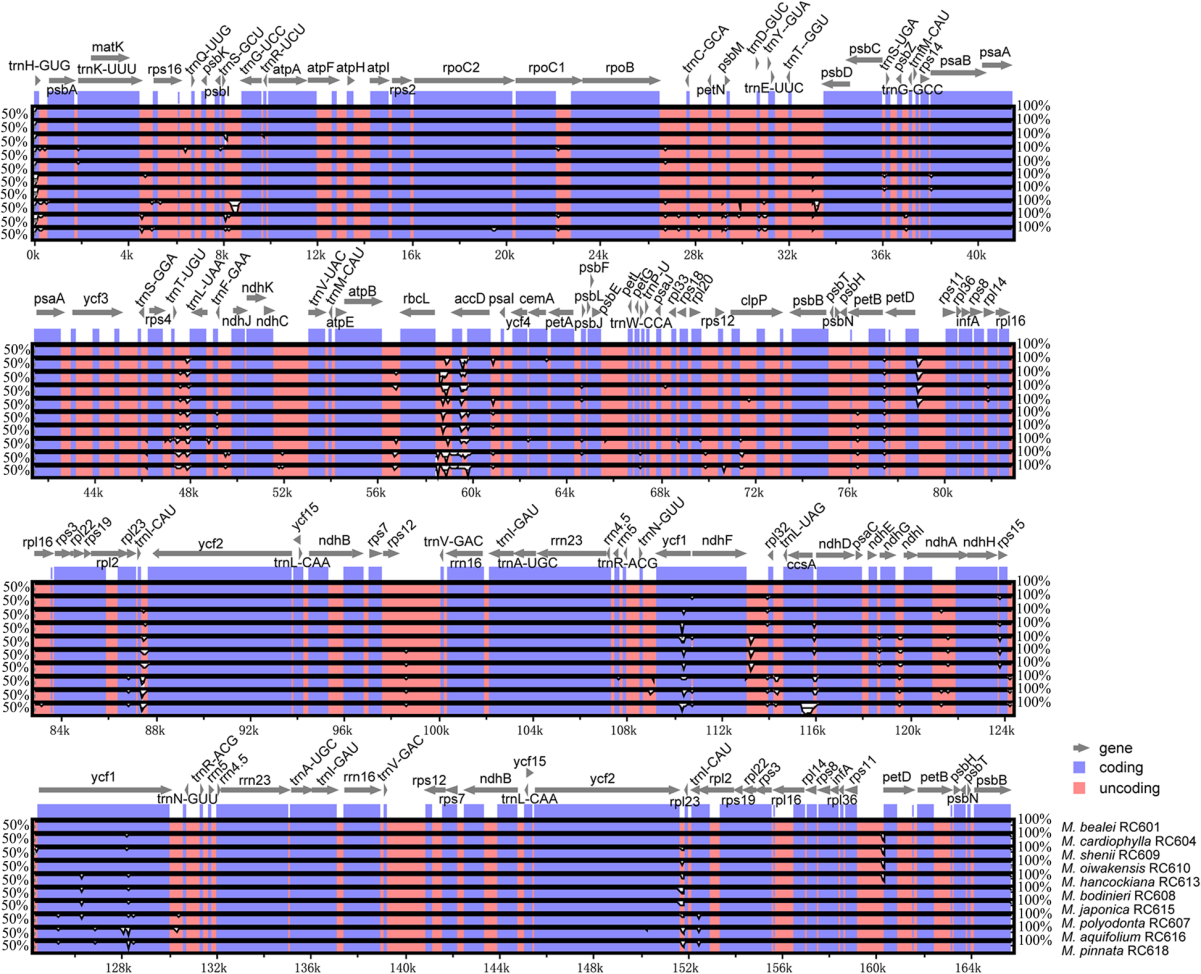
Visualization of alignment of ten plastomes of the species of *Mahonia* chloroplast genomes using mVISTA. *M*. *bealei* RC601 was used as a reference sequence. Blue represents coding regions, pink represents non-coding regions and gray arrows point at genes

Using an annotated plastid genome (*Mahonia bealei* RC601 [MZ158266]) as reference, we plotted two graphs for the overall sequence identity of ten *Mahonia* species and their outgroups using the program mVISTA (Figs. 3, S1). The results reveal that there are only slight variations within *Mahonia* plastid genomes. These variations are usually observed in the intergenic spacers (IGS) instead of coding-regions, which implies that coding regions are more conserved than non-coding regions (Fig. 3). The whole plastid genome of *M*. *bealei* RC601 [MZ158266] is also compared with those of *Berberis aristata* DC., *Ranzania japonica* (T. Itô ex Maxim.) T. Itô, *Gymnospermium kiangnanense* (P.L. Chiu) Loconte, *Leontice armeniaca* Boivin, *Caulophyllum robustum* Maxim. and *Nandina domestica* Thunb. However, the results reveal that there is a significant divergence in terms of sequence length, gene order and content among the genera related to *Mahonia* (Fig. S1). A large-scale IR expansion was found only in the plastomes of Berberideae, resulting in the additional duplication of 15 genes compared with typical angiosperm plastomes (Figs. S1, S2). The plastome size of *M*. *bealei* is about 165 kb and harbored more genes than other genera. Due to incomplete duplication of the normal copy, the gene *ycf1* across the IRb-SSC boundary is truncated to ca. 1346 bp and recognized as a pseudogene (ψ*ycf1*). Out of the three exons of the trans-splicing gene *rps12*, two are duplicated in the IR. Gene rearrangement is not observed within *Mahonia* plastid genomes (Fig. S3).

We compared the pairwise sequence distances and the number of nucleotide substitutions of 19 *Mahonia* species from 20 individuals. The highest level of pairwise sequence distance rate and the number of nucleotide substitutions (0.00546, 900 bp) is detected in the pair of *M*. *pinnata* and *M*. *japonica*. The lowest level (0, 0 bp) is observed between *M*. *bealei* RC601 [MZ158266] and *M*. *bealei* [MH795308] (Table S2).

### Contraction and expansion of IR regions

**Fig. 4:**
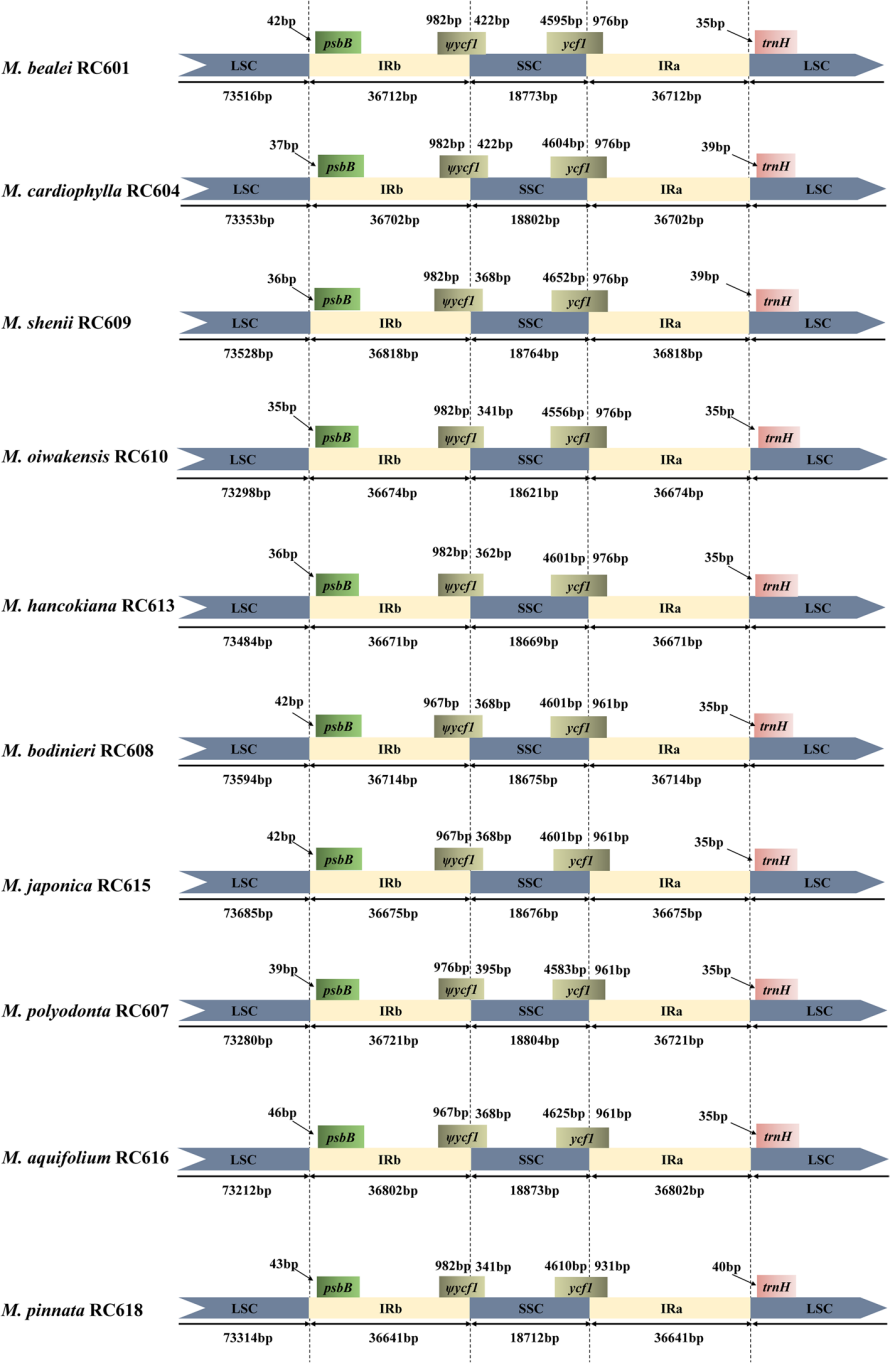
Comparison of the LSC, IR and SSC boundary regions of ten plastomes of the species of *Mahonia*

We compared the IR-SC boundaries among seven plastid genomes from different genera of Berberidaceae, and showed that the contraction and expansion of IR varied among different genera of Berberidaceae. Visualizing these whole plastomes, we observed a large expansion of IR in *Mahonia bealei*, as well as in *Berberis aristata*. As a result, about 12 kb corresponding to 15 genes (including *rps19*, *rpl22*, *rps3*, *rpl16*, *rpl14*, *rps8*, *infA*, *rpl36*, *rps11*, *petD*, *petB*, *psbH*, *psbN*, *psbT* and *psbB*) had suffered an additional duplication compared with the rest of the species we studied (Figs. S1, S2, Table S1). Thus, the IRb-LSC boundaries in these three species are located upstream of the *psbB* gene rather than located within the *rps19* gene, which is observed in the other four species we studied (*Caulophyllum robustum*, *Gymnospermium kiangnanense*, *Leontice armeniaca*, and *Nandina domestica*). The IRa-SSC and IRb-SSC boundaries are located within *ycf1* and *ycf1* pseudogenes (ψ*ycf1*), respectively. The *ndhF* genes, located downstream of the *ycf1* pseudogene, are 37 bp–540 bp away from the IRb-SSC boundaries. There are 7–77 bp from *trnH* genes to IRa-LSC boundaries (Fig. S2). In contrast, only slight shifts are observed in interspecies comparisons among ten *Mahonia* plastid genomes (Fig. 4).

### Identification of hypervariable regions

**Fig. 5:**
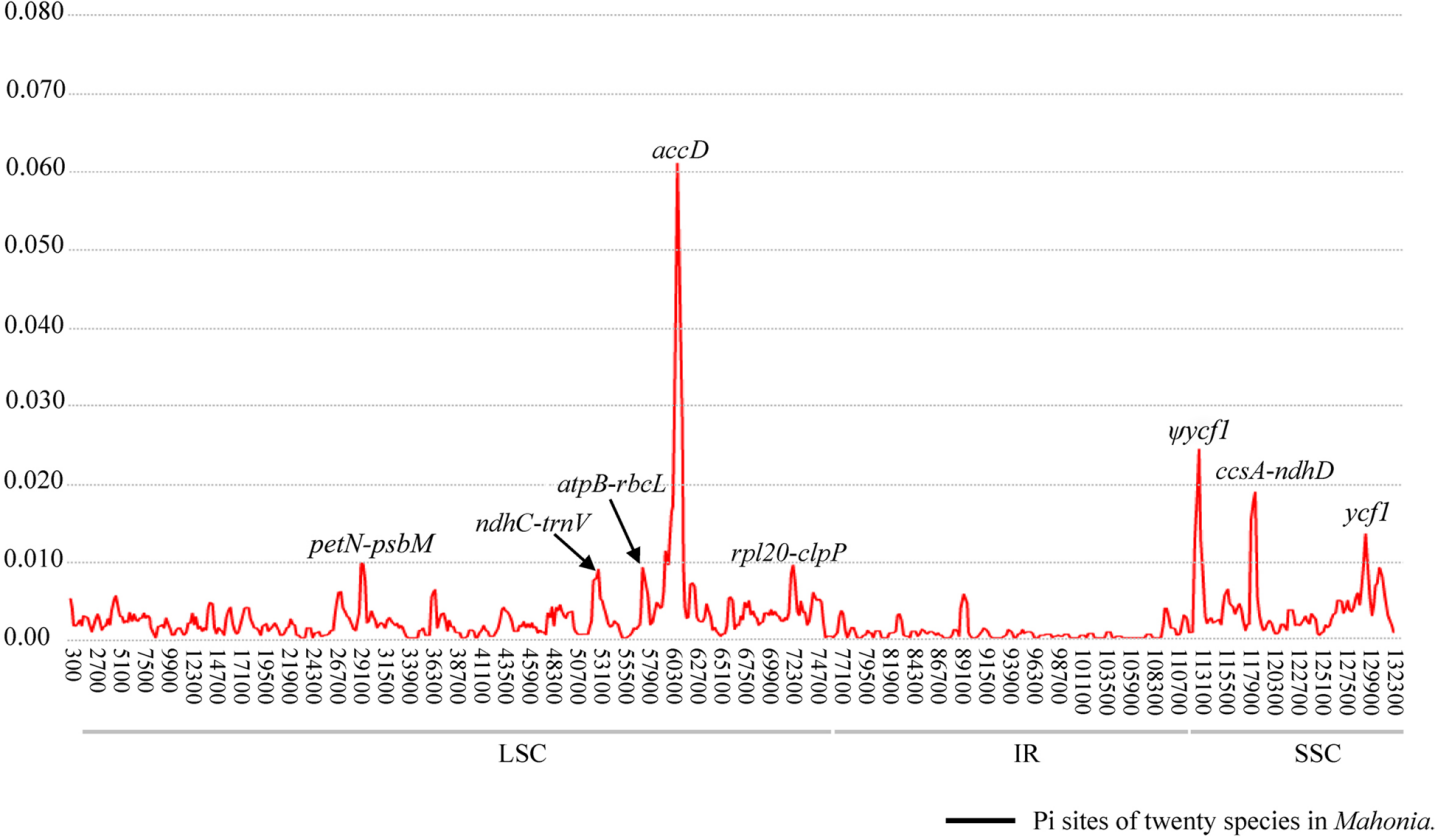
Sliding window analysis of the 20 plastomes of samples of *Mahonia*

Genome-wide sliding window analysis among 20 *Mahonia* individuals was performed in order to calculate the nucleotide diversity (Pi) values and identify the highly variable regions (mutational hotspots). The Pi values across the whole plastid genomes range from 0 to 0.06285 (mean = 0.00205), and the *accD* region exhibits the highest diversity level (Fig. 5). The eight most hypervariable regions (Pi > 0.008) were identified: five (*petN*–*psbM*, *ndhC*–*trnV*, *atpB*–*rbcL*, *accD*, *rpl20*–*clpP*) are located in the LSC, and the other three (*ycf1*, *ccsA–ndhD*, *ψycf1*) in the SSC. None was found in the IR (Fig. 5). The Pi values of the eight hypervariable regions we extracted range from 0.00311 to 0.0974 (Table 2).

**Table 2:** Sequence characteristics of eight highly variable regions among 20 plastomes

Region	Aligned length	Variable sites		Indels		Nucleotide diversity (Pi)
		No	%	No	Length range	
*petN*-*psbM*	661	27	4.08	24	1–9	0.0974
*ndhC*-*trnV*	1603	47	2.93	137	1–38	0.00580
*atpB*-*rbcL*	1134	11	0.970	405	1–171	0.00311
*accD*	1827	90	4.93	497	1–165	0.01391
*rpl20-clpP*	1429	41	2.87	55	1–13	0.00522
*ψycf1*	1425	43	3.02	118	1–54	0.00712
*ccsA-ndhD*	259	26	10.0	23	0–23	0.04237
*ycf1*	5820	114	1.96	352	1–78	0.00478

### Repeated sequence analyses

**Fig. 6:**
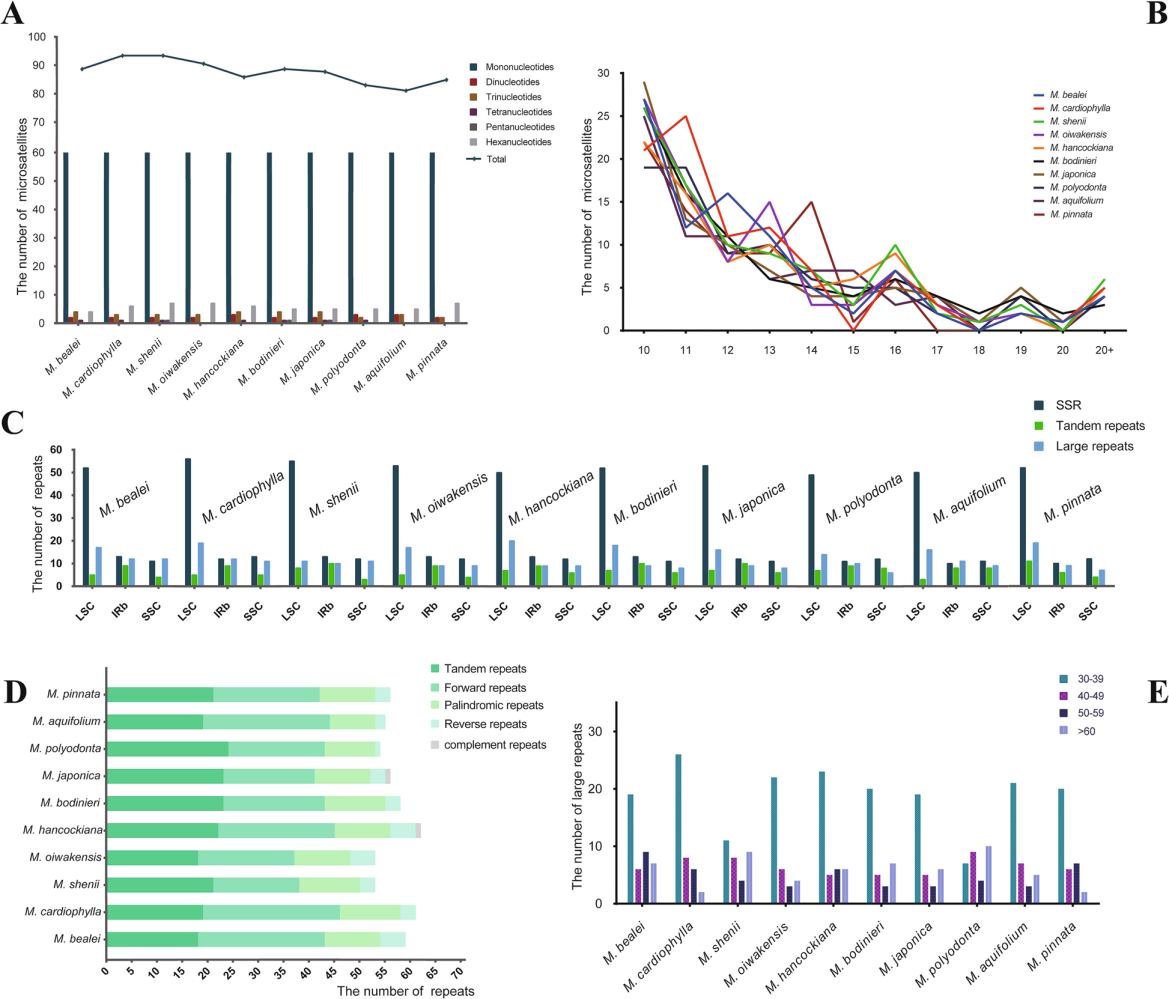
Analyses of SSRs and repeated sequences in plastomes of ten species of *Mahonia*. **A** Frequency of microsatellites by the length of repeated units. **B** Frequency of microsatellites by length. **C** Frequency of all repeats by location. **D** Numbers of five different types of repeats. **E** Frequency of four types of dispersed repeats by length

We used MISA to detect the simple sequence repeats (SSRs) among ten species of *Mahonia*. The number of SSRs in each *Mahonia* plastid genome varies from 81 in *M*. *aquifolium* to 94 in *M*. *cardiophylla* T.S. Ying & Boufford RC604 [MZ158269] and *M*. *shenii*. Within these SSRs, mononucleotides are the most abundant (86.1%), followed by hexanucleotides and then by dinucleotides and trinucleotides. In addition, tetranucleotides and pentanucleotides appear rarely in plastid genomes (Fig. 6A). The lengths of all the SSRs range from 10 to 28 bp, and a majority of the SSRs units possess 10 base pairs (Fig. 6B). Most of the SSRs are distributed in LSC regions, and the SSRs located in SSC regions and IR are nearly equal in size (Fig. 6C).

Overall, a total of 208 tandem repeats were identified within the ten *Mahonia* plastid genomes. Each plastome contains 18 to 24 tandem repeats (Fig. 6D). We recorded 359 dispersed repeats in the ten plastid genomes of this genus. Each plastome includes 30 to 42 dispersed repeats. The forward repeats account for the largest proportion of dispersed repeats (59.6%), followed by palindromic repeats and then by reverse repeats. Moreover, complement repeats are often absent, except for *M*. *japonica* (Thunb.) DC. RC615 [MZ158274] and *M*. *hancockiana* Takeda RC613 [MZ158273] (Fig. 6D). The most common types of dispersed repeats range from 30 to 39 bp in length (Fig. 6E).

### Phylogenetic analyses

**Fig. 7:**
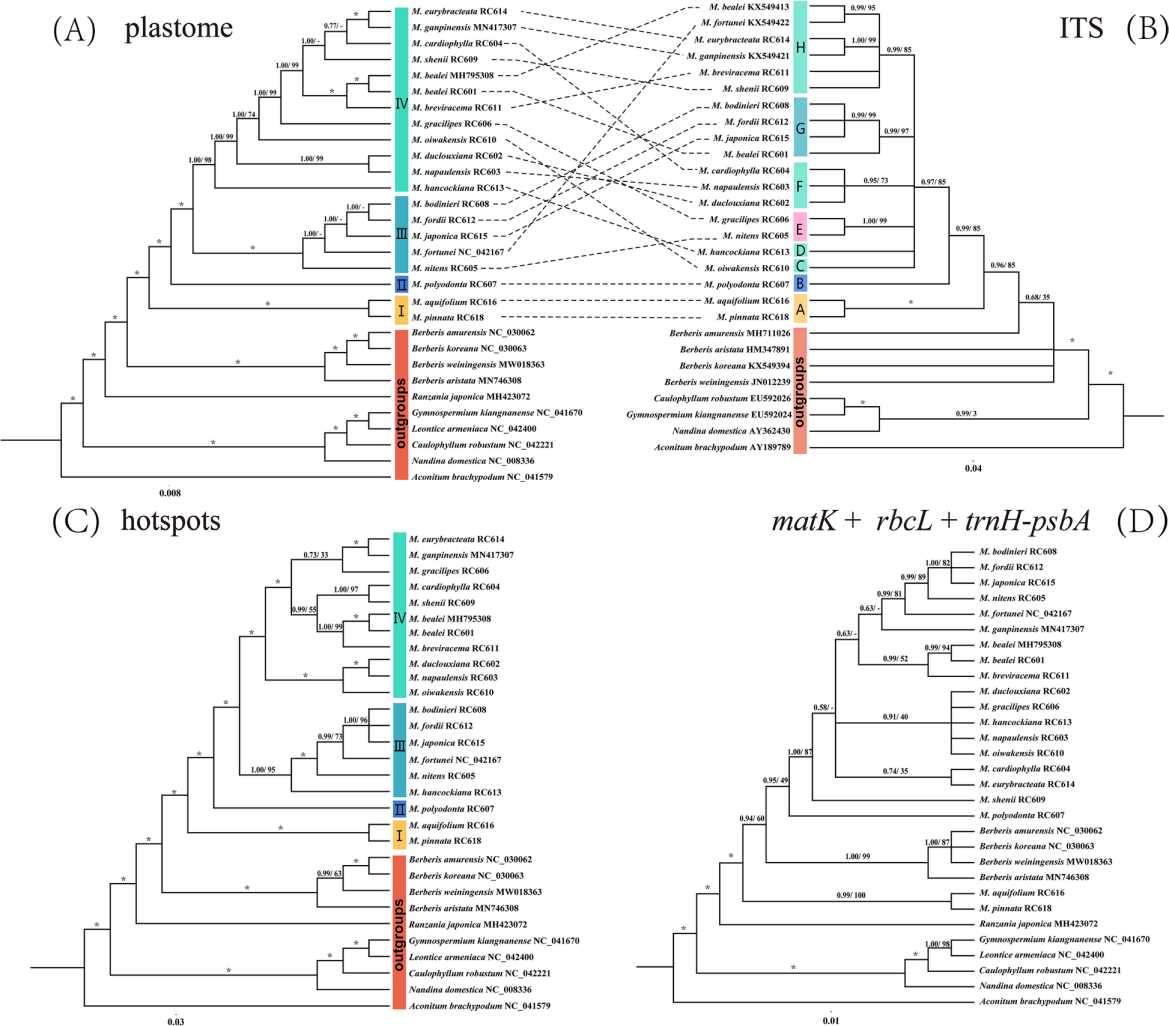
Phylogenetic relationships of *Mahonia* inferred from Bayesian inference (BI) and maximum likelihood (ML) based on four datasets. **A** complete plastomes. **B** ITS sequences. **C** hypervariable regions. **D** *rbcL* + *matK* + *trnH-psbA*. The support values above the branches show PP (posterior probability)/BS (bootstrap support), and asterisks indicate 1.00/100%. Dashes represent incongruences of BI and ML trees

In this study, nine alignment matrices were used to perform phylogenetic analyses using Bayesian inference (BI) and Maximum Likelihood (ML) method. The matrices consist of 20 ingroup accessions (*Mahonia*) and ten outgroup accessions. Notably, the ITS gene matrix includes only 28 samples, as the ITS sequences of *Leontice armeniaca* and *Ranzania japonica* are not available. The genus *Mahonia* is resolved as monophyletic and is sister to *Berberis* with strong support (bootstrap support (BS) ≥ 99%, posterior probabilities (PP) ≥ 0.99) across almost all trees (Figs. 7, S4, S5, S6). In the tree built using the complete plastid genome datasets, about 75 percent of the nodes are well supported (BS/PP = 99%/0.99). The phylogenetic trees exhibit that *Mahonia* species are grouped into four subclades. Subclade I comprises two species (*M*. *pinnata*, and *M*. *aquifolium*), which are both distributed in Western North America, while the species from the remaining subclades are native to East Asia. Subclade II contains a single species (*M*. *polyodonta* Fedde RC607 [MZ158279]). Subclade III consists of five species (*M*. *nitens* C.K. Schneid. RC605 [MZ158276], *M*. *fortunei* (Lindl.) Fedde [NC_042167], *M*. *japonica* (Thunb.) DC. RC615 [MZ158274], *M*. *fordii* C.K. Schneid. RC612 [MZ158271], and *M*. *bodinieri* Gagnep. RC608 [MZ158267]) with maximum PP support value (1.00). Regarding subclade III, the BI tree topology is not concordant with the ML tree topology. The remaining twelve individuals are gathered into subclade IV with high support values (BS/PP = 98%/1.00). Subclade I is the earliest-diverging lineage of *Mahonia*. Subclade IV is sister to subclade III, and together form a clade that is sister to subclade II (Fig. 7A).

To test the conflicting signals between plastomes and ITS sequences, both BI tree and ML tree based on ITS datasets were generated and together compared with the trees based on plastomes (Fig. 7B). As shown in Fig. 7B, the genus *Mahonia* is also recovered as a clade with moderate support (BS/PP = 0.96/85). Subclades I and II in the plastome tree are completely congruent with subclades A and B in the ITS tree. The tree topologies outside subclades I and II are incongruent. Indeed, the tree based on ITS sequences possessed the highest number of polytomies and could not provide any valuable information to resolve the infrageneric relationships. However, given that support values at internal nodes are much higher than the external, we can properly cluster the several subclades into subclades III and IV (in the plastome tree). Subclade III is largely identical to subclade G. Subclade IV gathers the remaining clades (C, D, F and H). Notably, the positions of *Mahonia fortunei* Chung 3342 [KX549421], *M*. *gracilipes* (Oliv.) Fedde RC606 [MZ158272] + *M*. *nitens* (subclade E) in the ITS tree are severely in conflict with the plastome tree.

It is noteworthy that the tree topology based on eight concatenated hypervariable regions is mainly identical to the whole plastid genome tree. Whereas the phylogenetic relationships within subclade III and IV based on whole plastid genomes (Fig. 7A) are not fully consistent with the topology from the hotspots (Fig. 7C). The trees based on concatenated *rbcL*, *matK*, and *trnH*-*psbA*, have the lowest phylogenetic resolution (Fig. 7D). Furthermore, none of the phylogenetic reconstructions based on the concatenated *rbcL*, *matK*, and *trnH*-*psbA* datasets provides any evidence for the monophyly of *Mahonia* (Fig. 7D).

Based on the five datasets (coding, non-coding, LSC, SSC, and IR regions) extracted from the plastid genomes, the overall topology is consistent with the topology retrieved from the complete plastid genome datasets. However, support values are high mostly at deep nodes (Fig. S4).

### Leaf morphological and micromorphological characteristics

**Fig. 8:**
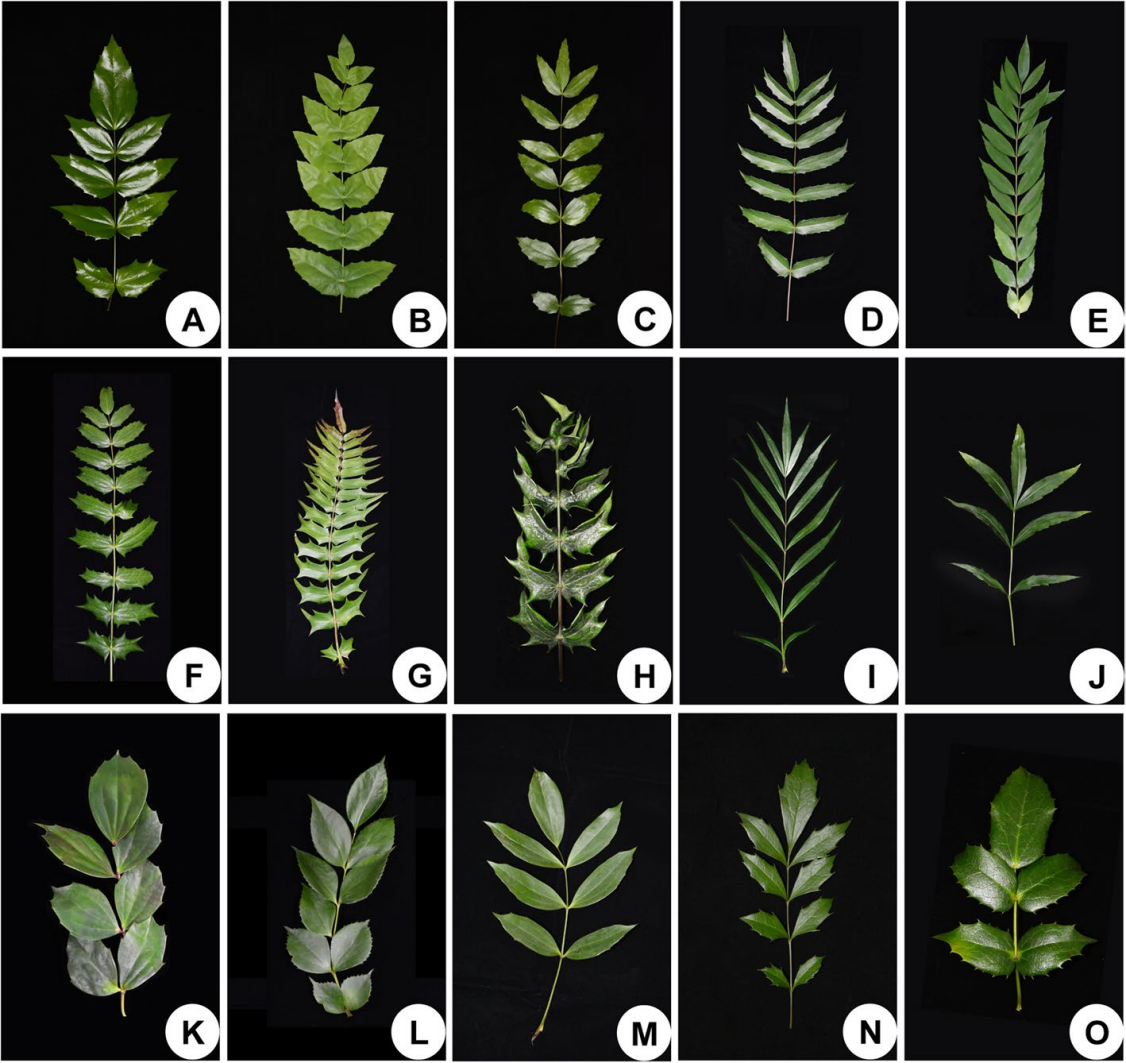
Morphological variations of compound leaves in *Mahonia*. **A** *M. fordii*. **B** *M. napaulensis*. **C** *M. hancockiana*. **D** *M*. *duclouxiana*. **E** *M. eurybracteata*. **F** *M. bordinieri*. **G** *M. oiwakensis*. **H** *M. cardiophylla*. **I** *M. eurybracteata* subsp. *ganpinensis*. **J** *M. fortunei*. **K** *M. nitens*. **L** *M. gracilipes*. **M** *M. shenii*. **N** *M. breviracema*. **O** *M. polyodonta*

**Fig. 9:**
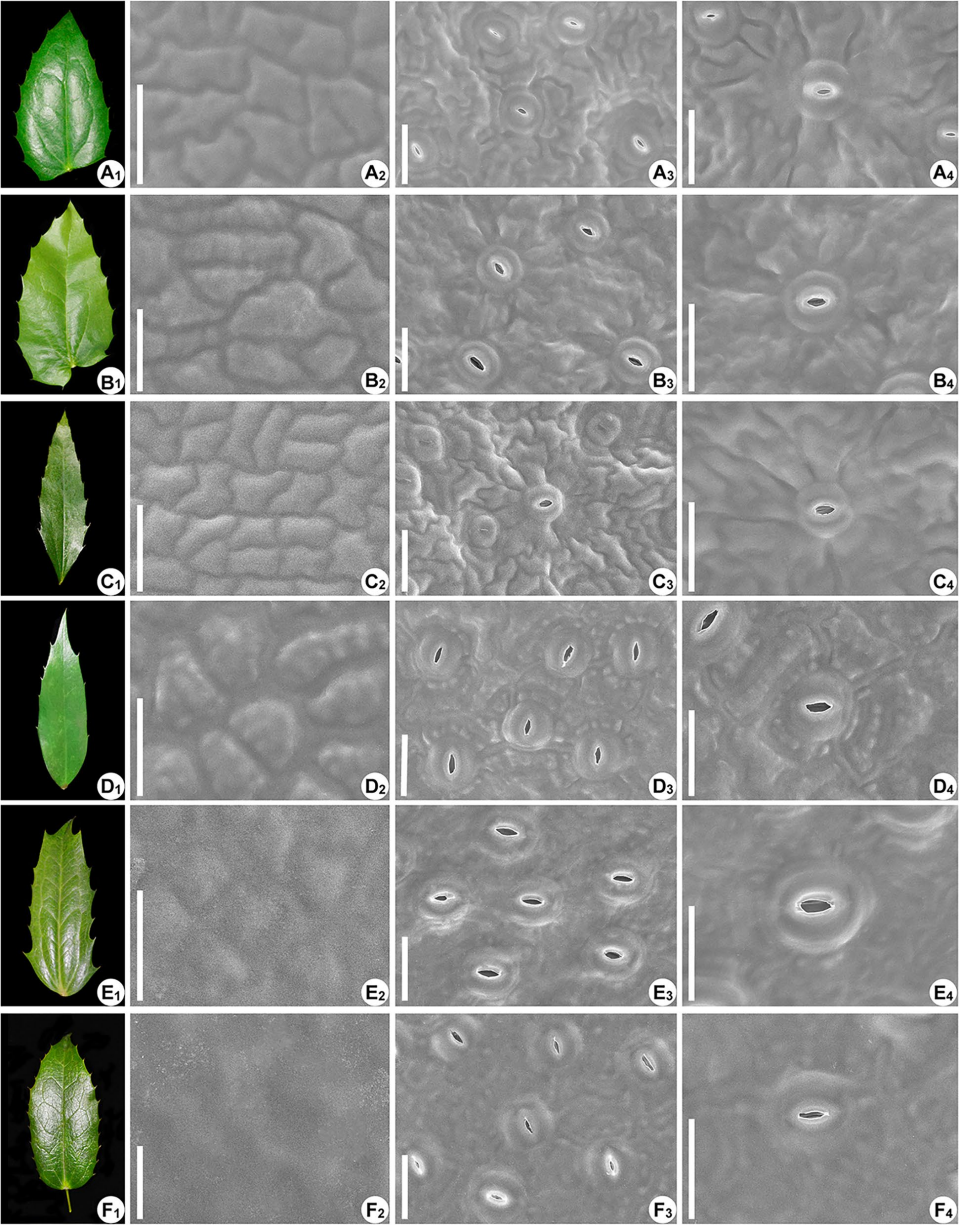
Characteristics of leaflets and epidermal surface. **A**_**1**_**–A**_**4**_ *M. bealei*. **B**_**1**_**–B**_**4**_ *M. napaulensis*. **C**_**1**_**–C**_**4**_ *M. fortunei*. **D**_**1**_**–D**_**4**_ *M. eurybracteata*. **E**_**1**_**–E**_**4**_ *M. bordinieri*. **F**_**1**_**–F**_**4**_ *M. polyodonta*. The images show leaflets, adaxial leaves, abaxial leaves and magnifying stomatal apparatus on the abaxial surface in each row from the left to right, respectively

The leaves of *Mahonia* are odd-pinnately compound. The adaxial surfaces of mature leaves are glossy for most species (Fig. 8). The leaflets show substantial diversity with respect to the number and shape among different species. Margins of each leaflet are variously toothed with coarse or fine spined serrations (Figs. 9A1–F1, S7A_1_–F_1_, S8A_1_–F_1_).

The adaxial surfaces of epidermal cells are almost convex (Figs. 9A2–D2, S7A_2_, B_2_, D_2_), slightly convex (Figs. 9E2, F2, S7C_2_, E_2_, S8A_2_, B_2_). Fewer upper surfaces are flat or nearly so (Figs. S7F_2_, S8C_2_). Seven species (*M*. *duclouxiana*, *M*. *cardiophylla*, *M*. *nitens*, *M*. *gracilipes*, *M*. *breviracema*, *M*. *eurybracteata* subsp. *ganpinensis*, and *M*. *pinnata*) show epicuticular waxes on the adaxial side of their leaves (Figs. S7D_2_–F_2_, S8C_2_–F_2_). On the abaxial surface of leaflets, cells with irregular shape and stomatal apparatus are found. The anticlinal walls of lower epidermal cells are either mostly inconspicuous or prominently sinuous, almost stellate in appearance (Figs. 9A3, C3, S7D_3_). Epidermal cells surrounding stomata are usually sunken, resulting in uneven lower epidermis of leaflets. Wax ornamentations in the form of strips is found on the abaxial surface of *M*. *hancockiana*, *M*. *breviracema* and *M*. *japonica* leaves (Figs. S7D_3_, F_3_, F_4_, S8B_3_, B_4_). All the leaves we observed are hypostomatic (Figs. 9, S7, S8). The stomata are anomocytic (Figs. S7D_4_, S8B_3_–D_3_, F_3_), cyclocytic (Fig. 9D3) or actinocytic (Figs. 9A3–C3, S7A_4_, B_4_, E_4_). In *M*. *bodinieri*, *M*. *polyodonta* and *M*. *nitens*, the abaxial surfaces of leaf epidermis are so flat that we could not detect the cell boundaries and determine the type of stomatal apparatus (Figs. 9E4, F4, S8E_4_).

## Discussion

### Comparative plastome of *Mahonia*

In the vast majority of flowering plants, complete plastomes share a similar structure comprising a large inverted repeat (IR), a large single copy (LSC) and a small single copy (SSC), respectively ~ 25 kb, ~ 87 kb and ~ 18 kb in length (e.g., *Diphylleia* Michaux, *Dysosma* Woodson, *Podophyllum* L., *Sinopodophyllum* Ying [(Berberidaceae)] [35]; *Maddenia* Hook. f. & Thoms, [36]; almost all genera of Styracaceae, [37]). The ebb and flow of IR are not unusual in evolutionary history [38]. IR recognition can display length divergence in angiosperm plastid genomes [39–41]. For instance, *Pelargonium transvaalense* R. Knuth possesses the largest known IR regions with ~ 88 kb in length [42]. More than 10 kb IR expansion was found in *Nicotiana acuminata* (Graham) Hook. (Solanaceae) [43]. In Trochodendrales, an IR expansion of about 4 kb was observed in both genera *Trochodendron* Siebold & Zucc. and *Tetracentron* Oliv. [20, 44]. A possible mechanism for these large and small IR expansions is double-strand DNA break and combination, and gene conversion, respectively [38].

Based on a chloroplast restriction site mapping study, Kim and Jansen [19] proposed that there was a large-scale (ca. 10 kb) IR expansion in the plastomes of Berberideae (*Berberis* and *Mahonia*). Ma et al. [20] conducted a comparative plastid genome analysis among four species of Ranunculales, finding that the genome size of *Mahonia bealei* was about 4.9–9.7 kb larger than the other three species (*Nandina domestica* [Berberidaceae], *Megaleranthis saniculifolia* Ohwi and *Ranunculus macranthus* Scheele [Ranunculaceae]). They inferred that a large IR expansion is the main cause of the significant increase in genome size in *Mahonia bealei* [20]. A similar 10 kb IR expansion has been described in *Ranzania japonica* [45] (GenBank ID: MG234280), although this result was not supported in other studies [46]. The plastome structure of *Ranzania japonica* is controversial and deserves further investigation. We present here the first comprehensive plastome analysis in *Mahonia* and show that there is a large IR expansion in the plastid genome of all species we investigated. Further intergeneric comparative plastome analyses have attested that a large-scale IR expansion was present in Berberideae [19, 46].

Although sequences in IR regions are commonly well conserved in comparison with single-copy (SC) regions, the IR-SC junctions are relatively variable. As shown in Fig. S2, neither large (> 500 bp) expansions nor contractions are recorded in the plastome of *Mahonia* species, except within the junctions of IRb-LSC. The IRb regions deeply expand into LSC regions reaching 10 kb in length, resulting in the IRb-LSC boundaries being located upstream of the gene *psbB*. For many angiosperm plastomes, the IRb-LSC boundaries are located in the gene *rps19* [36, 37, 47]. Previous studies have concluded that these variations at IR-SC boundaries can provide more information for elucidating the evolutionary patterns of closely related species [48] and selecting potential phylogenetic molecular markers [49].

In *Mahonia*, as SSRs and dispersed repeats show abundant variations among different species, they could be developed into molecular markers in the future.

### Phylogenetic analysis

In all phylogenetic trees we reconstructed (Figs. 7, S4, S5, S6), and except for the one based on the concatenation of the three common DNA barcodes (Fig. 7D), the monophyletic genus *Mahonia* was shown to be sister to *Berberis* with high support values, corroborating the results of previous studies [1, 2, 8]. Furthermore, in the plastome tree (Fig. 7A), the relationships among most clades are well resolved (PP = 1.00) implying the great power of using complete plastomes to address intractable phylogenetic relationships. Plastid phylogenomics of the family Berberidaceae [16] and of its different subordinate taxa have been studied in depth, including Podophylloideae [35], *Epimedium* [6], *Berberis* and *Mahonia* [2, 50]. Given the phylogeny topologies with strong support values, these results demonstrated the power of plastid phylogenomics for improving plastome-based phylogeny, investigating early-divergent events, and conducting taxonomic and plastome evolution analyses. Hsieh et al. [2] used 93 plastomes representing all 19 genera of Berberidaceae to resolve the long-standing disputable taxonomic issues of Berberidaceae. They also paid attention to the phylogeny and plastome structure of the tribe Berberideae, corroborating the considerable topological discordance between nrDNA and plastomes. Our phylogenomic analysis of the genus *Mahonia* based on more representative taxon sampling than previous studies, provides valuable genetic resources and improves our understanding of the relationships among phylogenetically challenging groups.

Determining the discordance between the topologies generated based on plastome and nuclear DNA has profound significance for clarifying the evolutionary events and evaluating the current phylogenetic frameworks generated by plastome datasets [51, 52]. In our study, we find significant discordance throughout the topologies of plastome tree and ITS tree in particular at deep nodes (Figs. 7A, B). Some nodes unexpectedly clustered with strong support values (e.g., subclades G and E in the ITS tree). These conflicts may be ascribed to ancestral hybridization events and/or incomplete lineage sorting [18, 52]. Exploring the source of discordant relationships is challenging especially in the hyper-diverse taxa, since radiations create opportunities for the evolutionary processes abovementioned [53]. Besides, we observed that the distribution of morphological characters is more congruent with the nuclear-based topology than with the plastome-based topology. Focusing on the ITS tree, the leaflets in subclade H exhibit a continuous morphological transition from linear to elliptic with several fine spined serrations at margins (Figs. 9C1, D1, S7D_1_, E_1_, S8A_1_). The leaflets of the two species *M*. *gracilipes* and *M*. *nitens* in subclade E show highly similar shapes, which are distinctive from the leaf shapes of all species we investigated (Figs. S8E_1_, F_1_). These results indicate that nuclear datasets may have broader implications for morphological character evolution, hybridization and/or incomplete lineage sorting.

Standard DNA barcodes have been shown to often lack sufficient variable characters and thus often fail to discriminate species among many lineages [50, 54–56]. Chen et al. [8] added two hypervariable plastid genes (*accD* and *ndhF*) and combined them with ITS, *rbcL*, *matK*, *trnH*-*psbA*. The combined ITS and plastid DNA dataset was used to conduct phylogenetic analyses, revealing that this kind of dataset could significantly improve the intergeneric resolution but had rarely power to address the interspecific phylogenetic relationships. In this paper, the concatenated universal DNA barcodes (*rbcL* + *matK* + *trnH*-*psbA*) expectedly failed to resolve the complex phylogenetic relationships among the species of *Mahonia*. On the contrary, based on eight concatenated hypervariable regions, the phylogenetic trees show a similar topological structure with the topology of the plastome trees and possess high support values. This phenomenon indicates that these hypervariable regions yield adequate information to address complicated phylogenetic relationships at the species level. Nowadays, an increasing number of studies consider the hypervariable regions to be valuable and introduce clade-specific barcodes (also named special barcodes) for phylogenetic purposes and even for the purpose of quick identification of medicinal plants [35, 36, 50, 57, 58]. Establishing clade-specific barcodes is far from easy as it depends on a series of factors, including the cost of whole plastome sequencing and sophisticated analytical tools [50]. Based on our results, we believe that the development of clade-specific barcodes has significant implications for species identification and biodiversity conservation in evolutionarily complex taxa [59].

### An integrative method for distinguishing closely related species

Micromorphological characteristics are usually constant within species and could be used for detailed species identification [30, 33, 60–62]. Despite a high micromorphological similarity for vegetative and/or reproductive organs among closely related species, micromorphological characteristics, e.g., the structure of glandular trichomes (*Arnebia* and *Lithospermum* in Boraginaceae [63]), petal epidermal cell patterns (Berberidaceae [31]), palynological characters (*Sanguisorba* (Rosaceae) [56]), have exhibited great diagnostic value. For instance, the patterns of lemma epidermis are taxonomically discriminant and frequently used to elucidate the phylogenetic relationships among different genera of Poaceae [64–66]. The different leaf epidermal characters are congruent with the different clades in *Cinnamomum* (Lauraceae) retrieved by Huang et al. (2016) [67], implying phylogenetic significance [68]. In addition, Wu et al. (2010) performed a set of investigations about seed morphology (i.e., seed size, color and shape, seed coat ornamentations) for 24 species of *Mahonia* [30]. They found that although seed morphological characters are conserved at the genus level, they are diversified enough to enable the division of the genus *Mahonia* into nine types for further systematic studies. In our micromorphological study, the type of stomata, the shape of epidermal cells, the pattern of anticlinal walls and cuticular ornamentation show high diversity among different species. These characters could be regarded as complementary evidence, in addition to molecular data, to distinguish among species of *Mahonia*.

Given the efficiency and objectivity of molecular data, standard DNA barcodes are used as essential elements for discriminating plants [56]. However, standard DNA barcodes (ITS, concatenated *rbcL*, *matK*, and *trnH*-*psbA*) could not be used to distinguish the species in the genus of *Mahonia*, due to the limited diagnostic information.

In the era of NGS technology, an increasing number of research groups can afford the cost of whole plastid genome sequencing and then employ the data sets to resolve challenging phylogenetic relationships [55, 62, 69, 70]. In this context, we generated a robust phylogenetic framework for the species of *Mahonia* using plastome datasets. Complete plastid genomes encompass adequate sequence variations for detailed identification, but their sequencing encounters some problems: high sequencing cost, huge-scale datasets and sophisticated computational process.

Based on eight concatenated hypervariable regions, the topology of the phylogenetic trees we reconstructed is mostly congruent with the topology of the tree based on the whole plastomes. This indicates that these hypervariable regions have adequate information which is almost equal to the information contained in the whole plastome. We extracted and developed these hypervariable regions into special barcodes, which combine the advantages of standard DNA barcodes and whole plastome [36, 71]. However, molecular data including standard and special barcodes could possibly remain unsuccessful at distinguishing among closely related species, especially in young lineages and lineages hosting an evolutionary radiation.

Micromorphological evidence can be used to address the different alternatives in resolving the polytomies in the tree built based on the special barcodes. For instance, the phylogenetic analysis could not differentiate the three species (*M*. *bordinieri*, *M*. *fordii*, *M*. *japonica*) from each other (Fig. 7C). However, we find that the adaxial surfaces of the epidermal cells of *M*. *bordinieri*, *M*. *fordii* and *M*. *japonica* are slightly convex, convex, and waxy, respectively (Figs. 9E2, S7A_2_, F_2_). In addition, the shape of epidermal cells of *M*. *bordinieri* and *M*. *fordii* is subquadrate and irregular, respectively. We could not determine the cell shape of *M*. *japonica* because of its invisible or obscure cell boundaries. On the abaxial surface of leaflets, we found actinocytic stomata in *M*. *fordii*. Several epidermal cells get organized in the form of a rosette around the stomata (Fig. S7A_4_). Anomocytic stomata and annular stripe were observed on the abaxial leaflets of *M*. *japonica* (Fig. S7F_4_). The epidermal cells on the abaxial surface of *M*. *bodinieri* appear to be considerably flat, which is obviously different from the other two species (Fig. 9E4). Therefore, these epidermal features could be used to distinguish the closely related species. Given the absence of wax ornamentations on the adaxial leaf surface of *M*. *bodinieri* and *M*. *fordii* compared to *M*. *japonica*, we suspect that the first two species above-mentioned might have a closer relationship.

Also, this study contributes micromorphological evidence to resolve polytomies in the ITS tree (Fig. 7B). The leaflets of *M*. *breviracema* are distinctly different from the leaflets of *M*. *shenii* regarding surface convexity. The former is convex with wax ornamentation (Fig. S7D_2_), while the latter is slightly convex (Fig. S8A_2_). The distinction indicates that *M*. *breviracema* may be closer to *M*. *bealei* and *M*. *fortunei*, and *M*. *shenii* may be closer to the *M*. *eurybracteata* RC614 [MZ158270] and *M*. *eurybracteata* subsp. *ganpinensis* (Fig. 7B). We can also apply micromorphological traits to differentiate *M*. *napaulensis*, *M*. *duclouxiana* and *M*. *cardiophylla* clearly (Figs. 9B2, S8C_2_, D_2_). The epidermal cells on the adaxial surface of leaves in *M*. *napaulensis* are convex without wax ornamentation, whereas the other two have substantial wax ornamentations in the form of stripes. More detailed comparison reveals that the leaf surfaces of *M*. *duclouxiana* are much flatter than the leaf surfaces of *M*. *cardiophylla*. However, we prefer to consider a closer relationship between *M*. *napaulensis* and *M*. *duclouxiana* based on the similar shape of leaflets (Figs. 9B1, S8C_1_).

## Conclusion

Based on the integration of molecular data from hypervariable regions and epidermal characters of leaflets, we can distinguish all the species we investigated within the genus *Mahonia*. Although our sampling for next-generation sequencing is not extensive enough to delimit species boundaries clearly, our results shed a light on the taxonomic, phylogenetic, and evolutionary analysis of the genus *Mahonia*. For further investigations, on the basis of a more comprehensive sampling, we propose an integrative method based on special barcodes and broader macroscopical evidence (e.g., morphological, micromorphological, anatomical and even cytological characteristics) to distinguish closely related species. Furthermore, genetically variable hotspots could be developed as clade-specific barcodes for efficient and rapid species identification especially in medicinal plants. It deserves ongoing and concerted efforts of the "barcode" research community to build a comprehensive system for accurately identifying plant species.

## Methods

### Taxon sampling, DNA extraction and next-generation sequencing

Fresh leaves from adult plants were collected from Sichuan, Yunnan, and Guizhou provinces of China and immediately dried using silica gel (Table 3). Liang Zhao from Herbarium of Northwest A&F University (WUK) undertook the formal identification of the vouchers and the plant materials used in our study. Voucher specimens of the plant materials we collected have been deposited in WUK. Dried leaves of four species of *Mahonia* (*M*. *oiwakensis* Hayata RC610 [MZ158277], *M*. *japonica* (Thunb.) DC. RC615 [MZ158274], *M*. *aquifolium* (Pursh) Nutt. RC616 [MZ158265] and *M*. *pinnata* (Lag.) Fedde RC618 [MZ158278]) were taken from voucher specimens of the Herbarium of Northwest A&F University (WUK). The 17 species sampled represented all the subclades of *Mahonia* and these species are from East Asia, Western North America and Europe, where it was inferred to be the center of diversity for *Mahonia* [8]. For this study, complete plastomes were obtained from 17 species of *Mahonia*. In addition, we obtained from GenBank plastomes of three *Mahonia* species (*M*. *eurybracteata* subsp. *ganpinensis* (H. Lév.) Fedde, GenBank ID: MN417307, *M*. *fortunei*, GenBank ID: NC_042167, and *M*. *bealei*, GenBank ID: MH795308) and plastomes for ten outgroup species to conduct subsequent comparative and phylogenetic analyses (Table 3). Total genomic DNAs were extracted from dried leaves using Cetyltrimethylammonium Bromide (CTAB) method [72] and sequenced using the Illumina Miseq platform (Illumina, San Diego, California, USA) at the Beijing Genomics Institute (BGI). Paired-end sequence reads have been trimmed to remove low-quality reads and adapter sequences using Trimmomatic v0.40 [73].

**Table 3:** Voucher information and GenBank accession numbers for *Mahonia* and outgroups

Taxon	Location	Date	Herbarium	Voucher	Identifier	Accession	SRA Accession	Alt.(m)	Longitude (E)	Latitude (N)
M. bealei*	China:YunNan	2020	WUK	RC601	Liang Zhao	MZ158266	SRR14460604	1539 m	103°17′31.00''E	29°34′2.24''N
M. duclouxiana*	China:GuiZhou	2020	WUK	RC602	Liang Zhao	MZ086770	SRR14429474	1107 m	106°41′48.03''E	26°32′7.89''N
M. napaulensis*	China:YunNan	2020	WUK	RC603	Liang Zhao	MZ158275	SRR14460603	1539 m	103°17′31.00''E	29°34′2.24''N
M. cardiophylla*	China:SiChuan	2020	WUK	RC604	Liang Zhao	MZ158269	SRR14460596	-	-	-
M. nitens*	China:SiChuan	2020	WUK	RC605	Liang Zhao	MZ158276	SRR14460595	-	-	-
M. gracilipes*	China:SiChuan	2020	WUK	RC606	Liang Zhao	MZ158272	SRR14460594	-	-	-
M. polyodonta*	China:SiChuan	2020	WUK	RC607	Liang Zhao	MZ158279	SRR14460593	-	-	-
M. bodinieri*	China:GuiZhou	2020	WUK	RC608	Liang Zhao	MZ158267	SRR14460592	1107 m	106°41′48.03''E	26°32′7.89''N
M. shenii*	China:GuiZhou	2020	WUK	RC609	Liang Zhao	MZ158280	SRR14460591	1107 m	106°41′48.03''E	26°32′7.89''N
M. oiwakensis*	China:YunNan	1952	WUK	RC610	Liang Zhao	MZ158277	SRR14460590	2400 m	-	**-**
M. breviracema*	China:YunNan	2020	WUK	RC611	Liang Zhao	MZ158268	SRR14460589	1539 m	103°17′31.00''E	29°34′2.24''N
M. fordii*	China:YunNan	2020	WUK	RC612	Liang Zhao	MZ158271	SRR14460602	1539 m	103°17′31.00''E	29°34′2.24''N
M. hancockiana*	China:YunNan	2020	WUK	RC613	Liang Zhao	MZ158273	SRR14460601	1539 m	103°17′31.00''E	29°34′2.24''N
M. eurybracteata*	China:SiChuan	2020	WUK	RC614	Liang Zhao	MZ158270	SRR14460600	-	-	-
M. japonica*	China:ShaanXi	1959	WUK	RC615	Liang Zhao	MZ158274	SRR14460599	-	-	-
M. aquifolium*	United Kingdom	1976	WUK	RC616	Liang Zhao	MZ158265	SRR14460598	500 m	-	-
M. pinnata*	USA	2000	WUK	RC618	Liang Zhao	MZ158278	SRR14460597	-	-	-
*M. bealei*	-	-	-	-	-	MH795308	-	-	-	-
*M. ganpinensis*	-	-	-	-	-	MN417307	-	-	-	-
*M. fortunei*	-	-	-	-	-	NC_042167	-	-	-	-
*Berberis koreana*	-	-	-	-	-	NC_030063	-	-	-	-
*Berberis amurensis*	-	-	-	-	-	NC_030062	-	-	-	-
*Berberis weiningensis*	-	-	-	-	-	MW018363	-	-	-	-
*Berberis aristata*	-	-	-	-	-	MN746308	-	-	-	-
*Ranzania japonica*	-	-	-	-	-	MH423072	-			
*Leontice armeniaca*	-	-	-	-	-	NC_042400	-	-	-	-
*Gymnospermium kiangnanense*	-	-	-	-	-	NC_041670	-	-	-	-
*Caulophyllum robustum*	-	-	-	-	-	NC_042221	-	-	-	-
*Nandina domestica*	-	-	-	-	-	NC_008336	-	-	-	-
*Aconitum brachypodum*	-	-	-	-	-	NC_041579	-	-	-	-

Species with asterisks were collected by this study, whereas others were obtained from GenBank

### Plastome assembly, annotation and visualization

We obtained approximately 2 GB high-quality data for each sample. The quality-filtered reads were then subjected to de novo assembling with GetOrganelle [74] or NOVOPlasty v4.3 [75], using *Mahonia oiwakensis* (GenBank ID: MN735221) as a reference for assembly. The 17 newly assembled plastome sequences were deposited in GenBank. We also submitted all the raw sequence data to GenBank and obtained SRA accessions (Table 3).

We annotated the 17 assembled plastomes with default parameters using Plastid Genome Annotator [76] (PGA) and inspected the accuracy of annotations with the annotation results from GeSeq [77]. On the basis of the results from PGA, we corrected the errors using Geneious v11.0.2 [78]. We checked the annotations of tRNA using tRNAscan-SE v2.0 [79]. The circular plastome maps of *Mahonia* were plotted using online OGDRAW [80].

### Comparative genomic analysis

Plastomes of ten *Mahonia* species were selected for further comparative genomics analyses and also repeated sequence identification. The ten species were representative of the different clades of *Mahonia*. We aligned the ten representative plastomes of *Mahonia* using MAFFT v7.450 [81] and adjusted the boundaries in Geneious. Following the same procedure, we aligned seven plastomes from different genera of Berberidaceae for further comparison of intergeneric sequence identity (see the details from section Results). Then, the two aligned matrices were visualized using online mVISTA program [82] under Shuffle-LAGAN mode with default options for other parameters. In both cases, the reference sequence was *M*. *bealei* RC601 [MZ158266]. Gene rearrangement events in *Mahonia* were detected using Mauve v2.4.0 [83].

Using Geneious, we compared the construction of ten representative plastomes of *Mahonia* and seven plastomes from different genera in Berberidaceae mentioned above. The IR-SC boundaries of plastomes of the species of *Mahonia* and of the outgroup species were manually detected and plotted.

We employed DnaSP v5.10 [84] to detect the plastid genome divergence and parsimony informative sites among 20 individuals (19 species) of *Mahonia*. A sliding window analysis (window length = 600, step size = 200) allowed us to determine hypervariable regions and estimate the level of polymorphism for subsequent phylogenetic analyses.

### Repeated sequence identification

Microsatellites (SSRs) were identified by MISA [85] with the thresholds of ten repeated units, and 6, 5, 5, 5, 4 repeated units for mono-, di-, tri-, tetra-, penta-, and hexanucleotide SSRs, respectively. We used the online Tandem Repeats Finder [86] to find the tandem repeated sequences with the default settings. REPuter program [87] was used to identify the dispersed repeated sequences, including forward, reverse, complement, and palindromic repeats. The minimum repeated size and Hamming distance were set at 30 bp and three (i.e., 90% sequence identity), respectively.

### Phylogenetic analyses

Phylogenetic analyses were made based on 17 newly sequenced complete plastomes of *Mahonia* and 13 already published plastid genomes (three species from *Mahonia* and ten species from Berberidaceae and Ranunculaceae for outgroup species). We aligned the plastid genomes and ITS sequences using MAFFT. Phylogenies were reconstructed based on the following datasets: (1) complete plastid genomes; (2) large-single-copy (LSC) region; (3) small-single-copy (SSC) region; (4) one inverted repeat (IR); (5) coding sequences; (6) non-coding sequences; (7) ITS; (8) concatenated sequences of *matK*, *rbcL*, and *trnH*-*psbA*; and (9) concatenated sequences of eight identified hypervariable regions. We applied the Maximum Likelihood (ML) method and Bayesian inference (BI) for each of the nine datasets to reconstruct phylogenetic trees, respectively. The ML analysis was carried out using RAxML-HPC Black Box [88] on the Cyberinfrastructure for Phylogenetic Research (CIPRES) Science Gateway [89], with 1000 bootstrap replicates and a GTRGAMMA + I model to obtain support values. jModelTest [90] was utilized to compute the best-fit model using the Akaike information criterion (AICc) for each partition, which was also conducted at the CIPRES Science Gateway (Table S3). BI trees were generated with MrBayes v3.2 [91]. The Markov chain Monte Carlo (MCMC) analysis was run for 10,000,000 generations and sampled every 1,000 generations. The first 25% trees were discarded as burn-in. The remaining trees were used to estimate the consensus tree and the Bayesian posterior probabilities.

### Recording of morphological and micromorphological character states

Images of mature leaves were taken with a Nikon 7100 camera (Nikon, Japan). Fresh leaves were first fixed in FAA (methanol: acetic acid: ethanol: water = 10:5:50:35). Next, small leaf pieces were dehydrated in an increasing alcohol series and isoamyl acetate series, and then, critical-point dried in CO_2_ with a K850 critical-point dryer (EMITECH, Ashford, England). Leaf pieces were then mounted on stubs and sputter coated with gold–palladium using a JS-1600 sputter coater (HTCY, China). The materials were photographed with a Hitachi S-3400 scanning electron microscope (SEM, Hitachi, Japan) at 15 kV.

## Supplementary Information

**Additional file 1:** **Fig. S1.** Visualization of alignment of *M*. *bealei* RC601 and six outgroups. *Mahonia bealei *RC601 was used as a reference sequence. Blue represents coding regions, pink represents non-coding regions and gray arrows points at genes. **Fig. S2.** Comparison of the LSC, IR and SSC boundary regions of plastomes of *M*.* bealei* RC601 and six outgroups. **Fig. S3.** Structural variation between plastomes of ten species of *Mahonia* revealed by Mauve. **Fig. S4.** Phylogenetic relationships of *Mahonia* inferred from BI and ML based on six datasets. A complete plastomes. B coding regions. C large single copy region. D non-coding regions. E small single copy region. F inverted repeated region. The support values above the branches show PP (posterior probability)/BS (bootstrap support), and asterisks indicate 1.00/100%. Dashes represent incongruences of BI and ML trees. **Fig. S5.** Phylogenetic trees of *Mahonia* showed by branch lengths from BI based on four datasets. A complete plastomes. B ITS sequences. C hypervariable regions. D *rbcL+matK+trnH-psbA*. The support values above the branches show PP (posterior probability). Branches without values indicate 1.00. **Fig. S6.** Phylogenetic trees of *Mahonia* showed by branch lengths from ML based on four datasets. A complete plastomes. B ITS sequences. C hypervariable regions. D *rbcL+matK+trnH-psbA*. The support values above the branches show BS (bootstrap support). Branches without values indicate 100. **Fig. S7.** Characteristics of leaflets and epidermal surface. A_1_–A_4_ *M. fordii*. B_1_–B_4_ *M. oiwakensis*. C_1_–C_4_ *M. aquifolium*. D_1_–D_4_ *M. breviracema*. E_1_–E_4_ *M. eurybracteata *subsp.* ganpinensis*. F_1_–F_4_ *M. japonica.* The images show leaflets, adaxial leaves, abaxial leaves and magnifying stomatal apparatus on the abaxial surface in each row from the left to right, respectively. **Fig. S8.** Characteristics of leaflets and epidermal surface. A_1_–A_4_ *M. shenii*. B_1_–B_4_ *M. hancockiana*. C_1_–C_4_ *M. duclouxiana*. D_1_–D_4_ *M. cardiophylla*. E_1_–E_4_ *M. nitens*. F_1_–F_4_ *M. gracilipes.* The images show leaflets, adaxial leaves, abaxial leaves and magnifying stomatal apparatus on the abaxial surface in each row from the left to right, respectively. **Table S1.** Gene composition of the 20 complete *Mahonia* chloroplast genomes. **Table S2.** Numbers of nucleotide substitutions and pairwise sequence distance rate in *Mahonia *plastomes. **Table S3.** Akaike information criterion (AICc) selection results for nine datasets.

## Data Availability

All data generated or analyzed during this study are included in this published article and its supplementary materials. All Illumina data have been deposited in NCBI’s Sequence Read Archive (SRA). Raw sequence reads are available on NCBI in the BioProject PRJNA727409 and PRJNA727753 (SAMN19020769–SAMN19020785). These sequence data have been submitted to the GenBank databases under accession number MZ086770, MZ158265–MZ158280.

## Abbreviations

*s.l.*: Sensu lato
*s.s.*: Sensu stricto
nrDNA: Nuclear ribosomal DNA
ITS: Internal transcribed spacer
LSC: Large single copy
SSC: Small single copy
IR: Inverted repeat
tRNA: Transfer RNA
rRNA: Ribosomal RNA
SSRs: Simple sequence repeats
BI: Bayesian inference
ML: Maximum likelihood
PP: Posterior probability
BS: Bootstrap support

## References

[1] Yu CC, Chung KF. Why *Mahonia*? Molecular recircumscription of *Berberis**s*.*l*., with the description of two new genera, *Alloberberis* and *Moranothamnus*. Taxon. 2017;66(6):1371–92. 10.12705/666.6.

[2] Hsieh CL, Yu CC, Huang YL, Chung KF. *Mahonia* vs. *Berberis* unloaded: generic delimitation and infrafamilial classification of Berberidaceae based on plastid phylogenomics. Front Plant Sci. 2022;12:720171. 10.3389/fpls.2021.720171. 10.3389/fpls.2021.720171 PMC8770955 35069611

[3] Berberidaceae LH, Kubitzki K, Rohwer JG, Bittrich V (1993). The families and genera of vascular plants II.

[4] Ying TS. Berberidaceae. In: Flora of China Editoral Committee, editor. Flora Reipublicae Popularis Sinicae. vol. 29. Beijing: Science Press; 2001. p. 50–305.

[5] Wang W, Chen ZD, Liu Y, Li RQ, Li JH (2007). Phylogenetic and biogeographic diversification of Berberidaceae in the northern hemisphere. Syst Bot.

[6] Zhang YJ, Du LW, Liu A, Chen JJ, Wu L, Hu WM, et al. The complete chloroplast genome sequences of five *Epimedium* species: lights into phylogenetic and taxonomic analyses. Front Plant Sci. 2016;7:306. 10.3389/fpls.2016.00306. 10.3389/fpls.2016.00306 PMC4791396 27014326

[7] Ahrendt LWA. *Berberis* and *Mahonia*: a taxonomic revision. Bot J Linn Soc. 1961;57(369):1–410. 10.1111/j.1095-8339.1961.tb00889.x.

[8] Chen XH, Xiang KL, Lian L, Peng HW, Erst AS, Xiang XG, et al. Biogeographic diversification of *Mahonia* (Berberidaceae): implications for the origin and evolution of East Asian subtropical evergreen broadleaved forests. Mol Phylogenet Evol. 2020;151:106910. 10.1016/j.ympev.2020.106910. 10.1016/j.ympev.2020.106910 32702526

[9] China Pharmcopia Committee (2015). Pharmacopoeia of the People’s Republic of China.

[10] Müller K, Ziereis K, Gawlik I. The antipsoriatic *Mahonia**aquifolium* and its active constituents; II Antiproliferative activity against cell growth of human keratinocytes. Planta Med. 1995;61(1):74–5. 10.1055/s-2006-958005. 10.1055/s-2006-958005 7700998

[11] Ying JS, Boufford DE, Brach AR. *Mahonia*. In: Wu ZY, Raven PH, editors. Flora of China. vol. 19. Beijing: Science Press; St. Louis, MO: Missouri Botanical Garden Press; 2011. p. 214–8.

[12] Adhikari B, Milne R, Pennington RT, Särkinen T, Pendry CA. Systematics and biogeography of *Berberis**s*.*l*. inferred from nuclear ITS and chloroplast *ndhF* gene sequences. Taxon. 2015;64(1):39–48. 10.12705/641.21.

[13] Kim YD, Kim SH, Landrum LR. Taxonomic and phytogeographic implications from ITS phylogeny in *Berberis* (Berberidaceae). J Plant Res. 2004;117(3):175–82. 10.1007/s10265-004-0145-7. 10.1007/s10265-004-0145-7 15015081

[14] Kim YD, Kim SH, Kim CH, Jansen RK. Phylogeny of Berberidaceae based on sequences of the chloroplast gene *ndhF*. Biochem Syst Ecol. 2004;32(3):291–301. 10.1016/j.bse.2003.08.002.

[15] Terabayashi S (1985). The comparative floral anatomy and systematics of the Berberidaceae II. Systematic considerations. Acta Phytotax Geobot.

[16] Sun YX, Moore MJ, Landis JB, Lin N, Chen L, Deng T (2018). Plastome phylogenomics of the early-diverging eudicot family Berberidaceae. Mol Phylogenet Evol.

[17] Colin O, Hinsinger DD, Strijk JS. *Mahonia**lancasteri* (Berberidaceae), a new species originating from Sichuan (China) described from cultivation. Phytotaxa. 2021;482(1):45–54. 10.11646/phytotaxa.482.1.5.

[18] Huang J, Su T, Lebereton-Anberrée J, Zhang ST, Zhou ZK. The oldest *Mahonia* (Berberidaceae) fossil from East Asia and its biogeographic implications. J Plant Res. 2016;129(2):209–23. 10.1007/s10265-015-0775-y. 10.1007/s10265-015-0775-y 26691316

[19] Kim YD, Jansen RK (1998). Chloroplast DNA restriction site variation and phylogeny of the Berberidaceae. Am J Bot.

[20] Ma J, Yang BX, Zhu W, Sun LL, Tian JK, Wang XM. The complete chloroplast genome sequence of *Mahonia**bealei* (Berberidaceae) reveals a significant expansion of the inverted repeat and phylogenetic relationship with other angiosperms. Gene. 2013;528(2):120–31. 10.1016/j.gene.2013.07.037. 10.1016/j.gene.2013.07.037 23900198

[21] Hebert PDN, Ratnasingham S, de Waard JR (2003). Barcoding animal life: Cytochrome c oxidase subunit 1 divergences among closely related species. Proc R Soc Lond B.

[22] Kress WJ, Wurdack KJ, Zimmer EA, Weigt LA, Janzen DH (2005). Use of DNA barcodes to identify flowering plants. Proc Natl Acad Sci U S A.

[23] Hollingsworth ML, Andra Clark A, Forrest LL, Richardson J, Pennington RT, Long DG (2009). Selecting barcoding loci for plants: evaluation of seven candidate loci with species-level sampling in three divergent groups of land plants. Mol Ecol Resour.

[24] CBOL Plant Working Group. Comparative analysis of a large dataset indicates that internal transcribed spacer (ITS) should be incorporated into the core barcode for seed plants. Proc Natl Acad Sci U S A. 2011;108(49):19641–6. 10.1073/pnas.1104551108. 10.1073/pnas.1104551108 PMC3241788 22100737

[25] Carstens BC, Pelletier TA, Reid NM, Satler JD (2013). How to fail at species delimitation. Mol Ecol.

[26] Wiens JJ (2007). Species delimitation: new approaches for discovering diversity. Syst Biol.

[27] Terabayashi S. Studies in the morphology and systematics of Berberidaceae: II. Floral anatomy of *Mahonia**japonica* (Thunb.) DC. and *Berberis**thunbergii* DC. Acta Phytotax Geobot. 1978;29(1–5):106–18. 10.18942/bunruichiri.KJ00001078289.

[28] Terabayashi S (1985). Seedling morphology of the Berberidaceae. Acta Phytotax Geobot.

[29] Brückner C. 2000. Clarification of the carpel number in Papaverales, Capparales, and Berberidaceae. Bot Rev. 2000;66(2):155–307. 10.1007/BF02858151

[30] Wu JY, Qin HN, Xue DY, Zhou KX. Study on seed morphology of *Mahonia* (Berberidaceae). Guihaia. 2010;30(2):155–60.

[31] Su S, Zhao L, Ren Y, Zhang XH (2021). Diversity of petals in Berberidaceae: development, micromorphology, and structure of floral nectaries. Protoplasma.

[32] Baranova MA (1987). Historical development of the present classification of morphological types of stomates. Bot Rev.

[33] Wu D, Wang H, Lu JM, Li DZ. Comparative morphology of leaf epidermis in *Parnassia* (Parnassiaceae) from China. Acta Phytotaxon Sin. 2005;43(3):210–24. 10.1360/aps040099.

[34] Shah SN, Celik A, Ahmad M, Ullah F, Zaman W, Zafar M (2019). Leaf epidermal micromorphology and its implications in systematics of certain taxa of the fern family Pteridaceae from Northern Pakistan. Microsc Res Tech.

[35] Ye WQ, Yap ZY, Li P, Comes HP, Qiu YX (2018). Plastome organization, genome-based phylogeny and evolution of plastid genes in Podophylloideae (Berberidaceae). Mol Phylogenet Evol.

[36] Su N, Liu BB, Wang JR, Tong RC, Ren C, Chang ZY, et al. On the species delimitation of the *Maddenia* group of *Prunus* (Rosaceae): evidence from plastome and nuclear sequences and morphology. Front Plant Sci. 2021;12:2135. 10.3389/fpls.2021.743643. 10.3389/fpls.2021.743643 PMC8542774 34707629

[37] Cai CN, Ma H, Ci XQ, Conran JG, Li J. Comparative phylogenetic analyses of Chinese *Horsfieldia* (Myristicaceae) using complete chloroplast genome sequences. J Syst Evol. 2021;59(3):504–14. 10.1111/jse.12556.

[38] Goulding SE, Olmstead RG, Morden CW, Wolfe KH (1996). Ebb and flow of the chloroplast inverted repeat. Mol Gen Genet.

[39] Tsudzuki J, Nakashima K, Tsudzuki T, Hiratsuka J, Shibata M, Wakasugi T, et al. Chloroplast DNA of black pine retains a residual inverted repeat lacking rRNA genes: nucleotide sequences of *trnQ*, *trnK*, *psbA*, *trnI* and *trnH* and the absence of *rps16*. Mol Gen Genet. 1992;232(2):206–14. 10.1007/BF00279998. 10.1007/BF00279998 1557027

[40] Kim KJ, Lee HL. Complete chloroplast genome sequences from Korean ginseng (*Panax**schinseng* Nees) and comparative analysis of sequence evolution among 17 vascular plants. DNA Res. 2004;11(4):247–61. 10.1093/dnares/11.4.247. 10.1093/dnares/11.4.247 15500250

[41] Abdullah ShahzadiI, Mehmood F, Ali Z, Malik MS, Waseem S, et al. Comparative analyses of chloroplast genomes among three *Firmiana* species: identification of mutational hotspots and phylogenetic relationship with other species of Malvaceae Plant. Gene. 2019;19:100199. 10.1016/j.plgene.2019.100199.

[42] Weng ML, Ruhlman TA, Jansen RK. Expansion of inverted repeat does not decrease substitution rates in *Pelargonium* plastid genomes. New Phytol. 2017;214(2):842–51. 10.1111/nph.14375. 10.1111/nph.14375 27991660

[43] Shen GF, Chen K, Wu M, Kung SD. *Nicotiana* chloroplast genome. Mol Gen Genet. 1982;187(1):12–8. 10.1007/BF00384377.

[44] Sun YX, Moore MJ, Meng AP, Soltis PS, Soltis DE, Li JQ (2013). Complete plastid genome sequencing of Trochodendraceae reveals a significant expansion of the inverted repeat and suggests a paleogene divergence between the two extant species. PLoS ONE.

[45] Wang M, Chen Y, Hina F, Ohi-Toma T, Li P. The complete chloroplast genome of *Ranzania**japonica*, an endangered species native to Japan. Conserv Genet Resour. 2018;10(4):671–4. 10.1007/s12686-017-0898-7.

[46] Kim YD, Jansen RK (1994). Characterization and phylogenetic distribution of a chloroplast DNA rearrangement in the Berberidaceae. Plant Syst Evol.

[47] Yan MH, Fritsch PW, Moore MJ, Feng T, Meng AP, Yang J (2018). Plastid phylogenomics resolves infrafamilial relationships of the Styracaceae and sheds light on the backbone relationships of the Ericales. Mol Phylogenet Evol.

[48] Menezes APA, Resende-Moreira LC, Buzatti RSO, Nazareno AG, Carlsen M, Lobo FP, et al. Chloroplast genomes of *Byrsonima* species (Malpighiaceae): comparative analysis and screening of high divergence sequences. Sci Rep. 2018;8:2210. 10.1038/s41598-018-20189-4. 10.1038/s41598-018-20189-4 PMC5797077 29396532

[49] Do HDK, Kim C, Chase MW, Kim JH (2020). Implications of plastome evolution in the true lilies (monocot order Liliales). Mol Phylogenet Evol.

[50] Kreuzer M, Howard C, Adhikari B, Pendry CA, Hawkins JA. Phylogenomic approaches to DNA barcoding of herbal medicines: developing clade-specific diagnostic characters for *Berberis*. Front Plant Sci. 2019;10:586. 10.3389/fpls.2019.00586. 10.3389/fpls.2019.00586 PMC6527895 31139202

[51] Smith SA, Moore MJ, Brown JW, Yang Y (2015). Analysis of phylogenomic datasets reveals conflict, concordance, and gene duplications with examples from animals and plants. BMC Evol Biol.

[52] Stull GW, Soltis PS, Soltis DE, Gitzendanner MA, Smith S (2020). Nuclear phylogenomic analyses of asterids conflict with plastome trees and support novel relationships among major lineages. Am J Bot.

[53] Fontaine MC, Pease JB, Steele A, Waterhouse M, Neafsey DE, Sharakhov IV (2015). Extensive introgression in a malaria vector species complex revealed by phylogenomics. Science.

[54] Simmonds SE, Smith JF, Davidson C, Buerki S. Phylogenetics and comparative plastome genomics of two of the largest genera of angiosperms, *Piper* and *Peperomia* (Piperaceae). Mol Phylogenet Evol. 2021;163:107229. 10.1016/j.ympev.2021.107229. 10.1016/j.ympev.2021.107229 34129936

[55] Fu CN, Mo ZQ, Yang JB, Cai J, Ye LJ, Zou JY, et al. Testing genome skimming for species discrimination in the large and taxonomically difficult genus *Rhododendron*. Mol Ecol Resour. 2022;22(1):404–14. 10.1111/1755-0998.13479. 10.1111/1755-0998.13479 34310851

[56] Park I, Song JB, Yang SY, Choi G. A comprehensive study of the genus *Sanguisorba* (Rosaceae) based on the floral micromorphology, palynology, and plastome analysis. Genes. 2021;12(11):1764. 10.3390/genes12111764. 10.3390/genes12111764 PMC8618895 34828370

[57] Vaughn JN, Chaluvadi SR, Tushar T, Rangan L, Bennetzen JL (2014). Whole plastome sequences from five ginger species facilitate marker development and define limits to barcode methodology. PLoS ONE.

[58] Manzanilla V, Kool A, Nguyen Nhat L, Van Nong H, Le Thi ThuH, de Boer HJ. Phylogenomics and barcoding of *Panax*: toward the identification of ginseng species. BMC Evol Biol. 2018;18(1):44. 10.1186/s12862-018-1160-y. 10.1186/s12862-018-1160-y PMC5883351 29614961

[59] Bi Y, Zhang MF, Xue J, Dong R, Du YP, Zhang XH. Chloroplast genomic resources for phylogeny and DNA barcoding: a case study on *Fritillaria*. Sci Rep. 2018;8(1):1184. 10.1038/s41598-018-19591-9. 10.1038/s41598-018-19591-9 PMC5775360 29352182

[60] Kong HZ (2001). Comparative morphology of leaf epidermis in the Chloranthaceae. Bot J Linn Soc.

[61] Ren H, Pan KY, Chen ZD, Wang RQ (2003). Structural characters of leaf epidermis and their systematic significance in Vitaceae. Acta Phytotax Sin.

[62] Li QJ, Su N, Zhang L, Tong RC, Zhang XH, Wang JR, et al. Chloroplast genomes elucidate diversity, phylogeny, and taxonomy of *Pulsatilla* (Ranunculaceae). Sci Rep. 2020;10(1):19781. 10.1038/s41598-020-76699-7. 10.1038/s41598-020-76699-7 PMC7666119 33188288

[63] Park I, Yang SY, Song JH, Moon BC. Dissection for floral micromorphology and plastid genome of valuable medicinal *Borages**Arnebia* and Lithospermum (Boraginaceae). Front Plant Sci. 2020;11:606463. 10.3389/fpls.2020.606463. 10.3389/fpls.2020.606463 PMC7746654 33343605

[64] Thomasson JR (1978). Epidermal patterns of the lemma in some fossil and living grasses and their phylogenetic significance. Science.

[65] Ortúñez E, de la Fuente V. Epidermal micromorphology of the genus *Festuca* L. in the Iberian Peninsula. Plant Syst Evol. 2010;284(3):201–18. 10.1007/s00606-009-0248-7.

[66] Nobis M. Taxonomic revision of the *Stipa**lipskyi* group (Poaceae: *Stipa* section *Smirnovia*) in the Pamir Alai and Tian-Shan Mountains. Plant Syst Evol. 2013;299(7):1307–54. 10.1007/s00606-013-0799-5.

[67] Huang JF, Li L, van der Werff H, Li HW, Rohwer JG, Crayn DM, et al. Origins and evolution of cinnamon and camphor: a phylogenetic and historical biogeographical analysis of the *Cinnamomum* group (Lauraceae). Mol Phylogenet Evol. 2016;96:33–44. 10.1016/j.ympev.2015.12.007. 10.1016/j.ympev.2015.12.007 26718058

[68] Gang Z, Liu B, Rohwer JG, Ferguson DK, Yang Y. Leaf epidermal micromorphology defining the clades in *Cinnamomum* (Lauraceae). PhytoKeys. 2021;182:125–48. 10.3897/phytokeys.182.67289. 10.3897/phytokeys.182.67289 PMC8516828 34720625

[69] Nock CJ, Waters DLE, Edwards MA, Bowen SG, Rice N, Cordeiro GM (2011). Chloroplast genome sequences from total DNA for plant identification. Plant Biotechnol J.

[70] Coissac E, Hollingsworth PM, Lavergne S, Taberlet P (2016). From barcodes to genomes: extending the concept of DNA barcoding. Mol Ecol.

[71] Li XW, Yang Y, Henry RJ, Rossetto M, Wang YT, Chen SL (2015). Plant DNA barcoding: from gene to genome. Biol Rev.

[72] Doyle J, Doyle J (1987). A rapid DNA isolation procedure for small quantities of fresh leaf tissue. Phytochemistry.

[73] Bolger AM, Lohse M, Usadel B (2014). Trimmomatic: a flexible trimmer for Illumina sequence data. Bioinformatics.

[74] Jin JJ, Yu WB, Yang JB, Song Y, dePamphilis CW, Yi TS, et al. Getorganelle: a fast and versatile toolkit for accurate *de**novo* assembly of organelle genomes. Genome Biol. 2020;21(1):241. 10.1186/s13059-020-02154-5. 10.1186/s13059-020-02154-5 PMC7488116 32912315

[75] Dierckxsens N, Mardulyn P, Smits G. NOVOPlasty: *de**novo* assembly of organelle genomes from whole genome data. Nucleic Acids Res. 2017;45(4):e18. 10.1093/nar/gkw955. 10.1093/nar/gkw955 PMC5389512 28204566

[76] Qu XJ, Moore MJ, Li DZ, Yi TS (2019). PGA: a software package for rapid, accurate, and flexible batch annotation of plastomes. Plant Methods.

[77] Tillich M, Lehwark P, Pellizzer T, Ulbricht-Jones ES, Fischer A, Bock R (2017). GeSeq-Versatile and accurate annotation of organelle genomes. Nucleic Acids Res.

[78] Kearse M, Moir R, Wilson A, Stones-Havas S, Cheung M, Sturrock S (2012). Geneious Basic: An integrated and extendable desktop software platform for the organization and analysis of sequence data. Bioinformatics.

[79] Lowe TM, Chan PP (2016). tRNAscan-SE On-line: integrating search and context for analysis of transfer RNA genes. Nucleic Acids Res.

[80] Lohse M, Drechsel O, Kahlau S, Bock R (2013). OrganellarGenomeDRAW–a suite of tools for generating physical maps of plastid and mitochondrial genomes and visualizing expression data sets. Nucleic Acids Res.

[81] Katoh K, Standley DM (2013). MAFFT multiple sequence alignment software version 7: improvements in performance and usability. Mol Biol Evol.

[82] Frazer KA, Pachter L, Poliakov A, Rubin EM, Dubchak I (2004). Vista: computational tools for comparative genomics. Nucleic Acids Res.

[83] Darling AE, Mau B, Perna NT (2010). progressiveMauve: multiple genome alignment with gene gain, loss and rearrangement. PLoS ONE.

[84] Librado P, Rozas J (2009). DnaSP v5: a software for comprehensive analysis of DNA polymorphism data. Bioinformatics.

[85] Thiel T, Michalek W, Varshney RK, Graner A. Exploiting EST databases for the development and characterization of gene-derived SSR-markers in barley (*Hordeum**vulgare* L.). Theor Appl Genet. 2003;106(6):411–22. 10.1007/s00122-002-1031-0. 10.1007/s00122-002-1031-0 12589540

[86] Benson G (1999). Tandem repeats finder: a program to analyze DNA sequences. Nucleic Acids Res.

[87] Kurtz S, Choudhuri JV, Ohlebusch E, Schleiermacher C, Stoye J, Giegerich R (2001). REPuter: the manifold applications of repeat analysis on a genomic scale. Nucleic Acids Res.

[88] Stamatakis A (2006). RaxML-VI-HPC: maximum likelihood-based phylogenetic analyses with thousands of taxa and mixed models. Bioinformatics.

[89] Miller MA, Pfeiffer W, Schwartz T. Creating the CIPRES Science Gateway for inference of large phylogenetic trees. In: 2010 Gateway Computing Environments Workshop (GCE); 2010. New Orleans. p. 1–8.

[90] Darriba D, Taboada GL, Doallo R, Posada D (2012). jModelTest 2: more models, new heuristics and parallel computing. Nat Methods.

[91] Ronquist F, Teslenko M, van der Mark P, Ayres DL, Darling A, Höhna S (2012). Mrbayes 3.2: efficient bayesian phylogenetic inference and model choice across a large model space. Syst Biol.

