# Supplementary material for: Phylogenomics, plastome structure and species identification in Mahonia (Berberidaceae)
Source: BMC Genomics. 2022 Nov 23;23:766. doi: 10.1186/s12864-022-08964-0 (PMC9682747; doi:10.1186/s12864-022-08964-0)
Supplement: Supplementary file 1 — Additional file 1: Fig. S1. Visualization of alignment of M. bealei RC601 and six outgroups. Mahonia bealei RC601 was used as a reference sequence. Blue represents coding regions, pink represents non-coding regions and gray arrows points at genes. Fig. S2. Comparison of the LSC, IR and SSC boundary regions of plastomes of M. bealei RC601 and six outgroups. Fig. S3. Structural variation between plastomes of ten species of Mahonia revealed by Mauve. Fig. S4. Phylogenetic relationships of Mahonia inferred from BI and ML based on six datasets. A complete plastomes. B coding regions. C large single copy region. D non-coding regions. E small single copy region. F inverted repeated region. The support values above the branches show PP (posterior probability)/BS (bootstrap support), and asterisks indicate 1.00/100%. Dashes represent incongruences of BI and ML trees. Fig. S5. Phylogenetic trees of Mahonia showed by branch lengths from BI based on four datasets. A complete plastomes. B ITS sequences. C hypervariable regions. D rbcL+matK+trnH-psbA. The support values above the branches show PP (posterior probability). Branches without values indicate 1.00. Fig. S6. Phylogenetic trees of Mahonia showed by branch lengths from ML based on four datasets. A complete plastomes. B ITS sequences. C hypervariable regions. D rbcL+matK+trnH-psbA. The support values above the branches show BS (bootstrap support). Branches without values indicate 100. Fig. S7. Characteristics of leaflets and epidermal surface. A1–A4 M. fordii. B1–B4 M. oiwakensis. C1–C4 M. aquifolium. D1–D4 M. breviracema. E1–E4 M. eurybracteata subsp. ganpinensis. F1–F4 M. japonica. The images show leaflets, adaxial leaves, abaxial leaves and magnifying stomatal apparatus on the abaxial surface in each row from the left to right, respectively. Fig. S8. Characteristics of leaflets and epidermal surface. A1–A4 M. shenii. B1–B4 M. hancockiana. C1–C4 M. duclouxiana. D1–D4 M. cardiophylla. E1–E4 M. nitens. F1–F4 M. gracilipes. [file 12864_2022_8964_MOESM1_ESM.doc]

**Supplementary Figures and Tables**


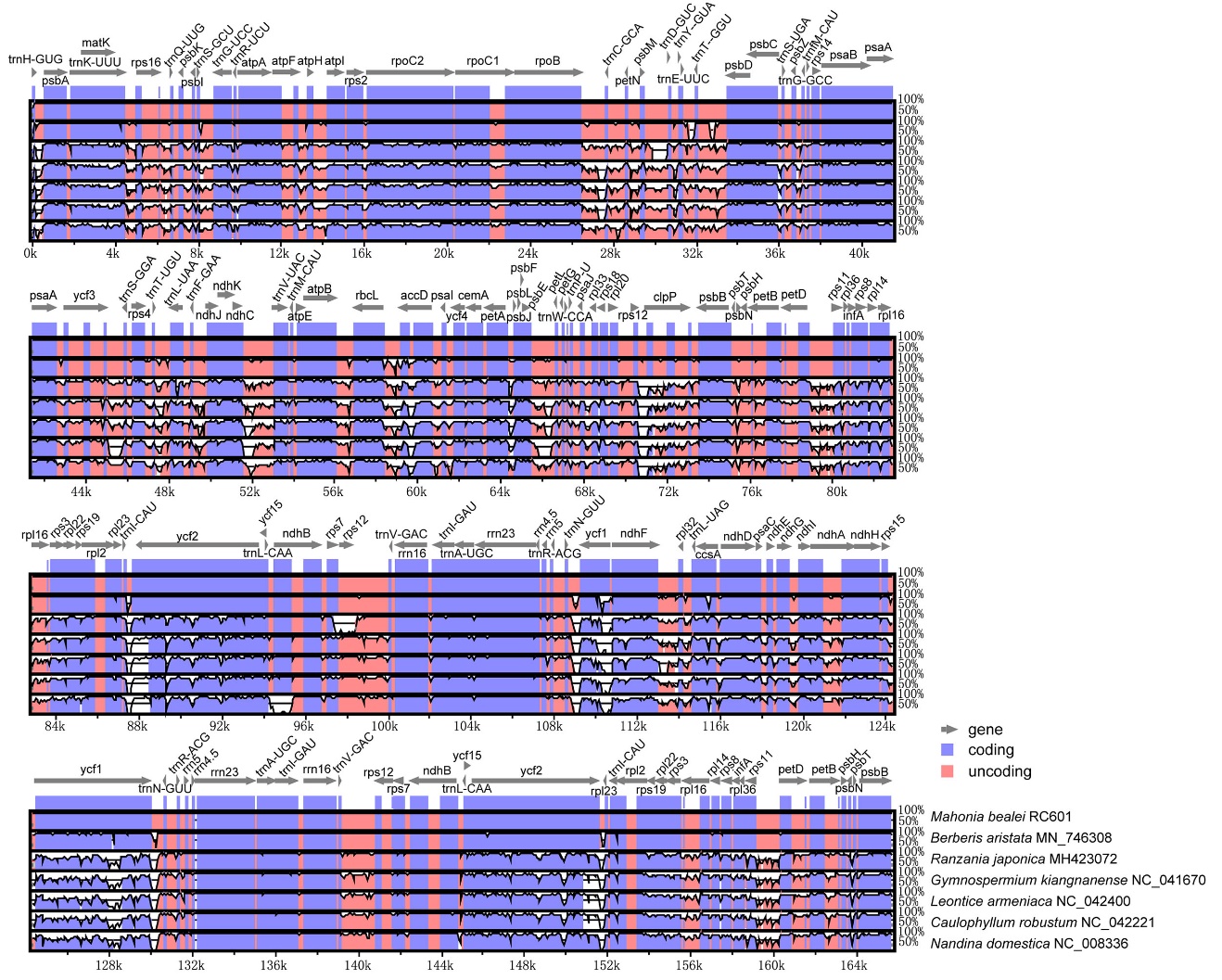


**Fig. S1** Visualization of alignment of *M*. *bealei* RC601 and six outgroups.

*Mahonia bealei* RC601 was used as a reference sequence. Blue represents coding regions, pink represents non-coding regions and gray arrows points at genes.


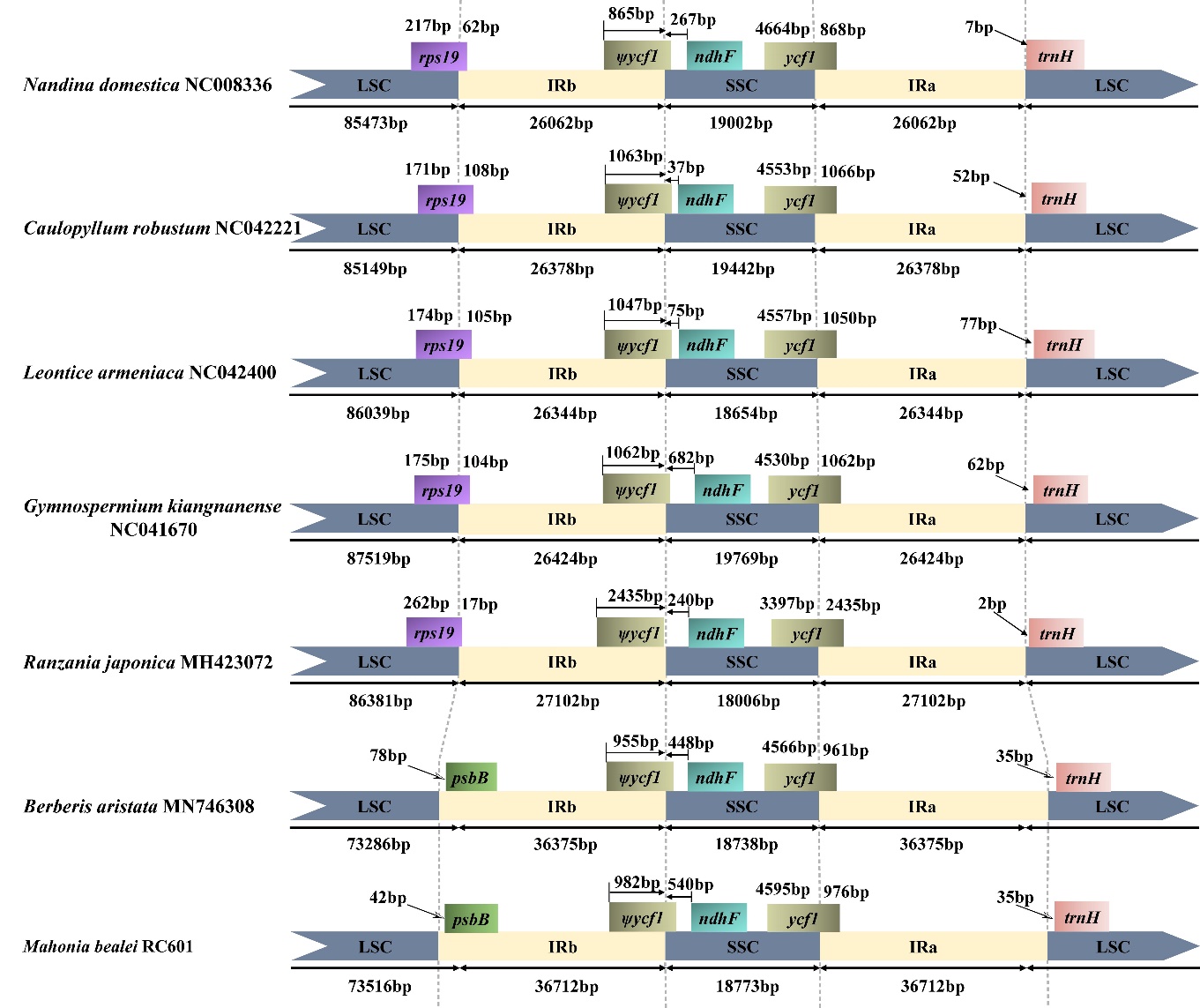


**Fig. S2** Comparison of the LSC, IR and SSC boundary regions of plastomes of *M*. *bealei* RC601 and six outgroups.


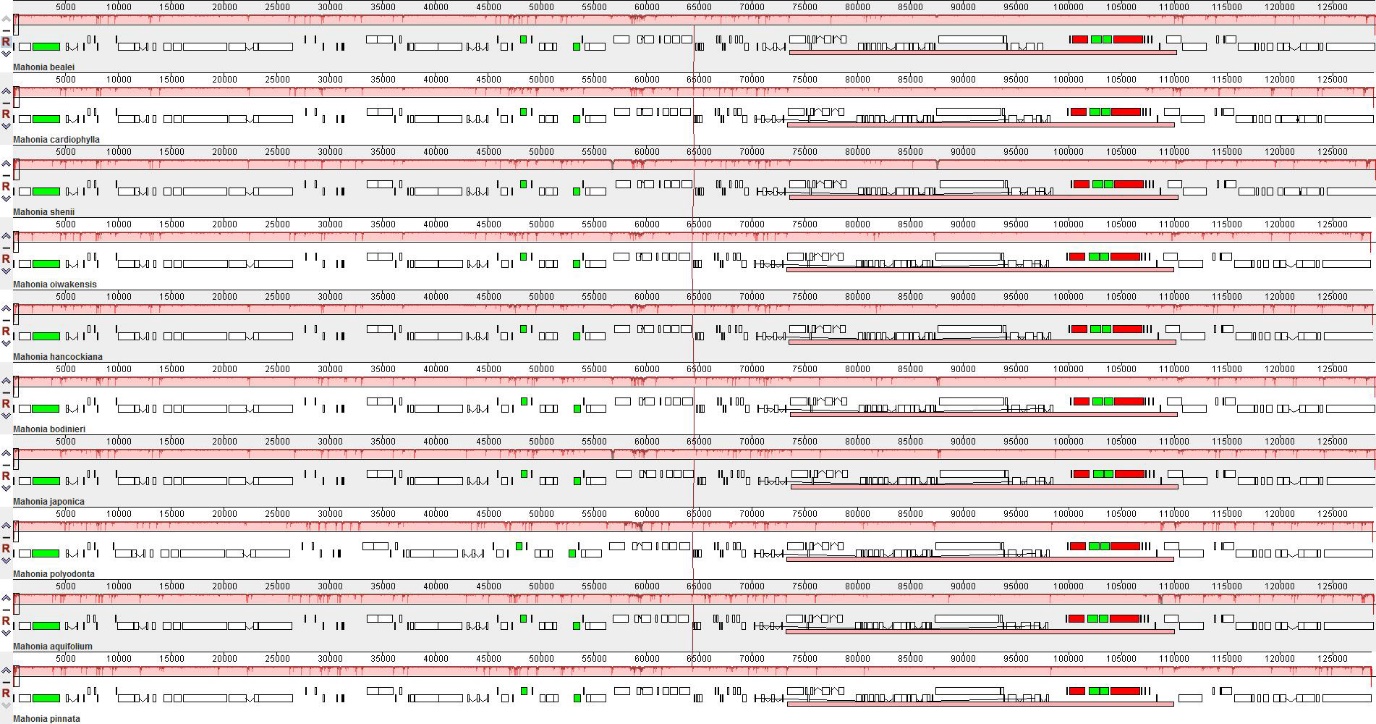


**Fig. S3** Structural variation between plastomes of ten species of *Mahonia* revealed by Mauve.


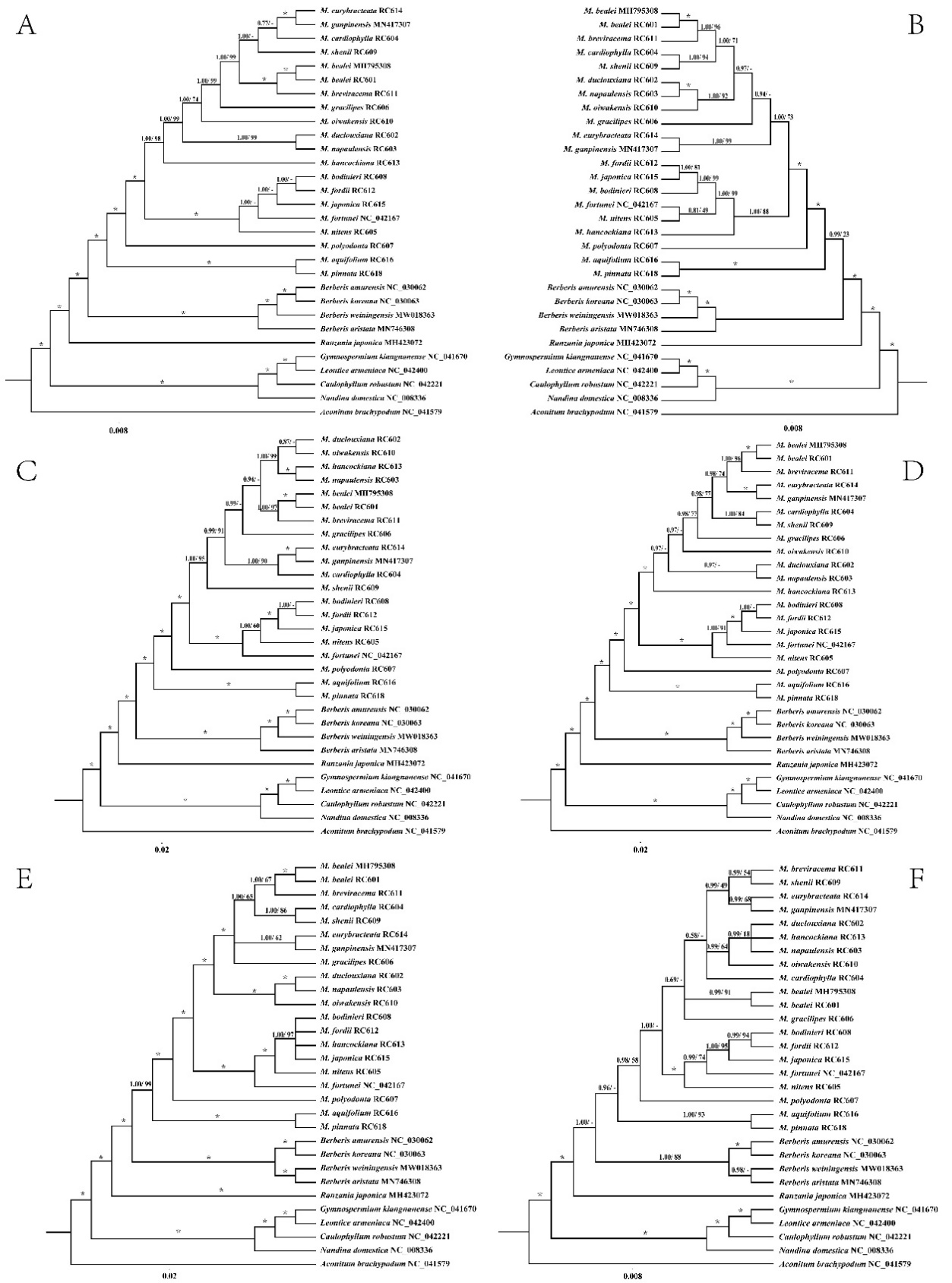


**Fig. S4** Phylogenetic relationships of *Mahonia* inferred from BI and ML based on six datasets.

**A** complete plastomes. **B** coding regions. **C** large single copy region. **D** non-coding regions. **E** small single copy region. **F** inverted repeated region. The support values above the branches show PP (posterior probability)/BS (bootstrap support), and asterisks indicate 1.00/100%. Dashes represent incongruences of BI and ML trees.


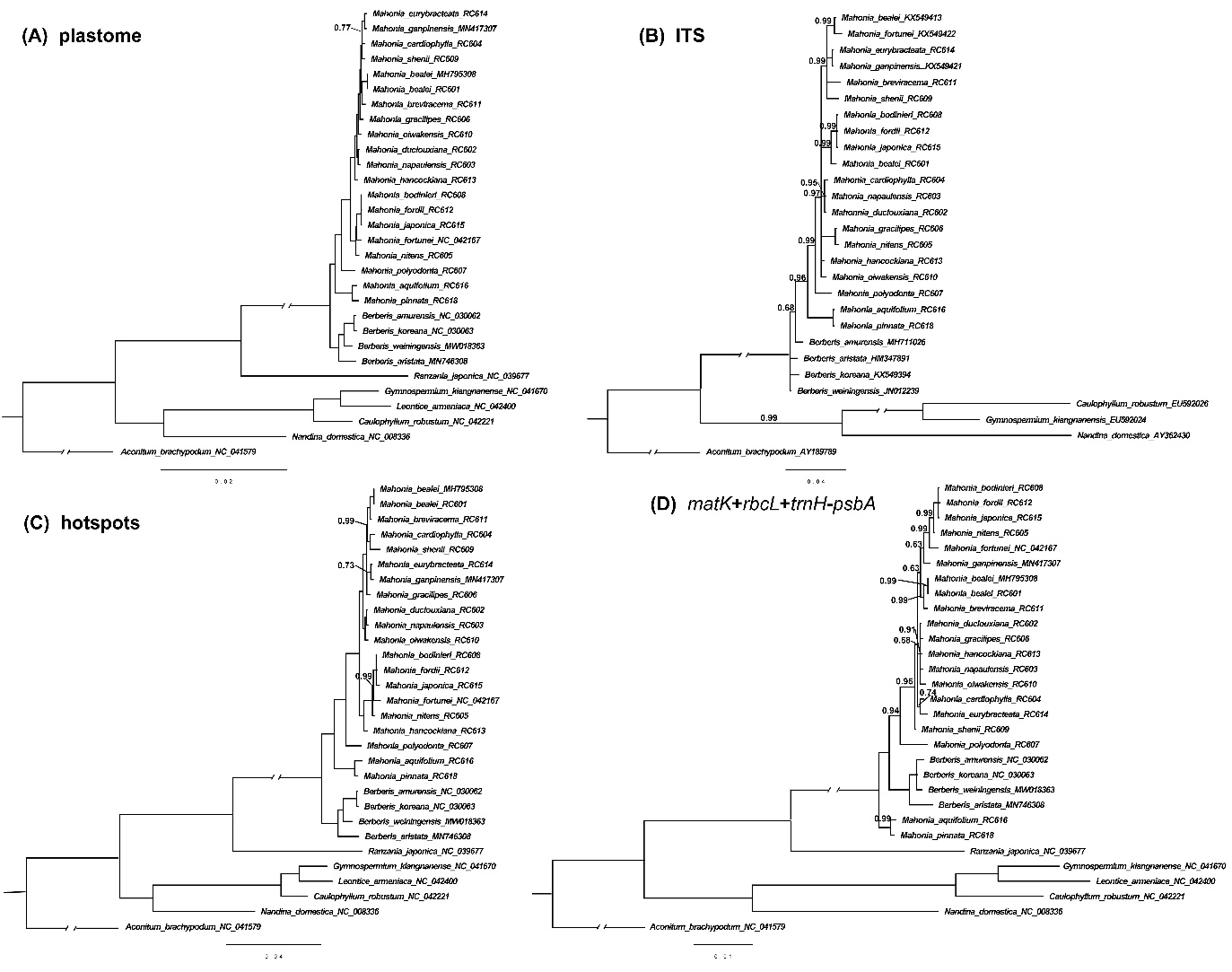


**Fig. S5** Phylogenetic trees of *Mahonia* showed by branch lengths from BI based on four datasets.

**A** complete plastomes. **B** ITS sequences. **C** hypervariable regions. **D** *rbcL+matK+trnH-psbA*. The support values above the branches show PP (posterior probability). Branches without values indicate 1.00.


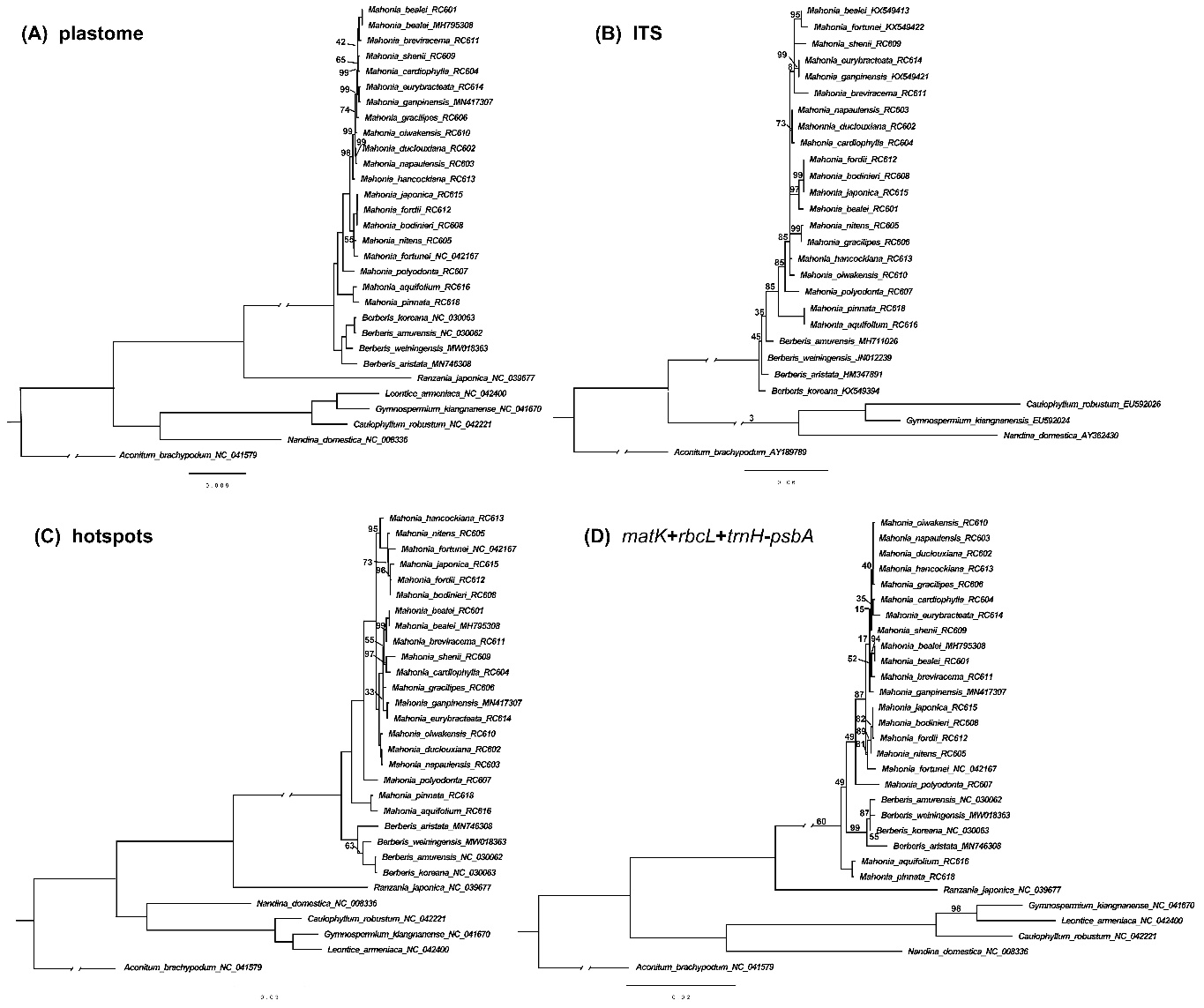


**Fig. S6** Phylogenetic trees of *Mahonia* showed by branch lengths from ML based on four datasets.

**A** complete plastomes. **B** ITS sequences. **C** hypervariable regions. **D** *rbcL+matK+trnH-psbA*. The support values above the branches show BS (bootstrap support). Branches without values indicate 100.


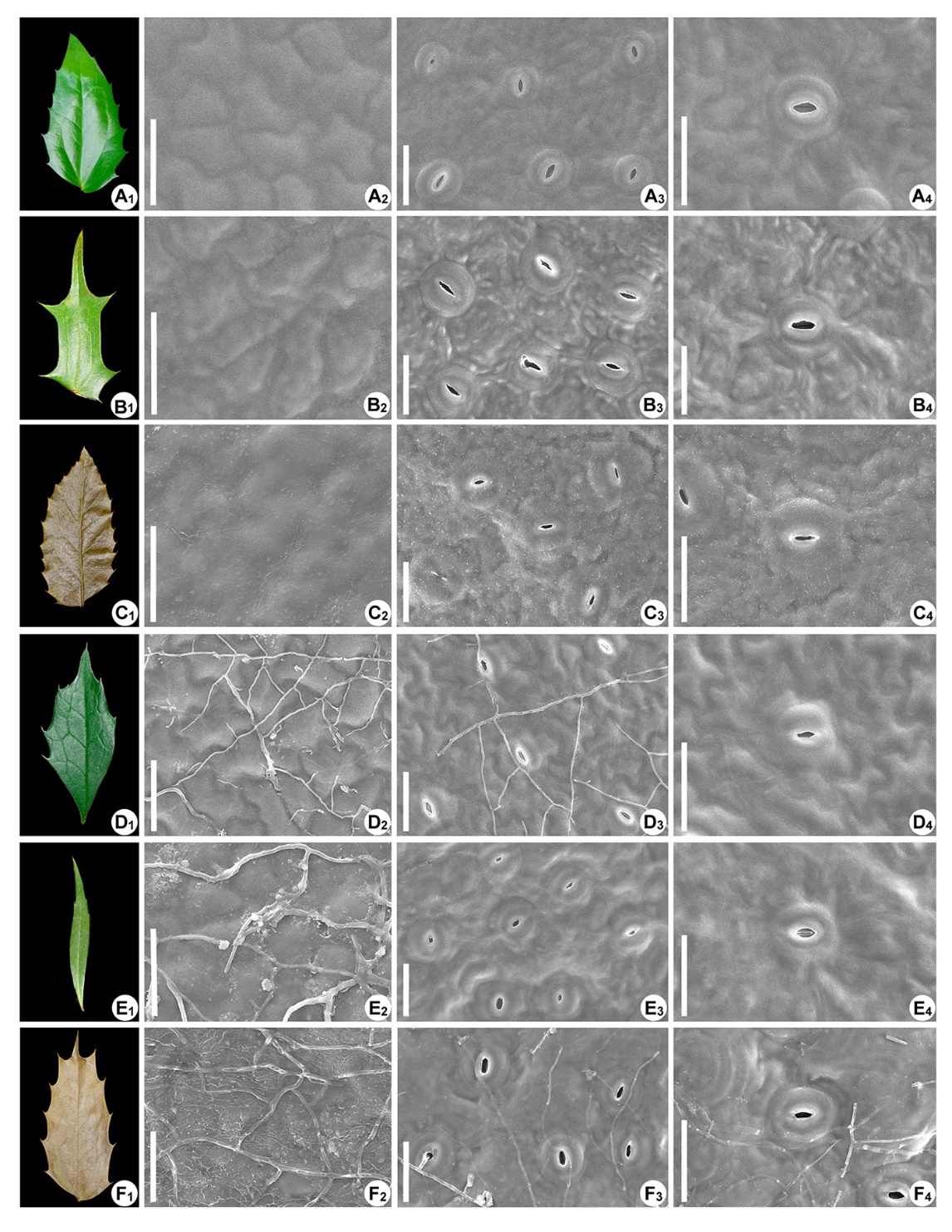


**Fig. S7** Characteristics of leaflets and epidermal surface.

**A_1_–A_4_** *M. fordii*. **B_1_–B_4_** *M. oiwakensis*. **C_1_–C_4_** *M. aquifolium*. **D_1_–D_4_** *M. breviracema*. **E_1_–E_4_** *M. eurybracteata* subsp. *ganpinensis*. **F_1_–F_4_** *M. japonica.* The images show leaflets, adaxial leaves, abaxial leaves and magnifying stomatal apparatus on the abaxial surface in each row from the left to right, respectively.


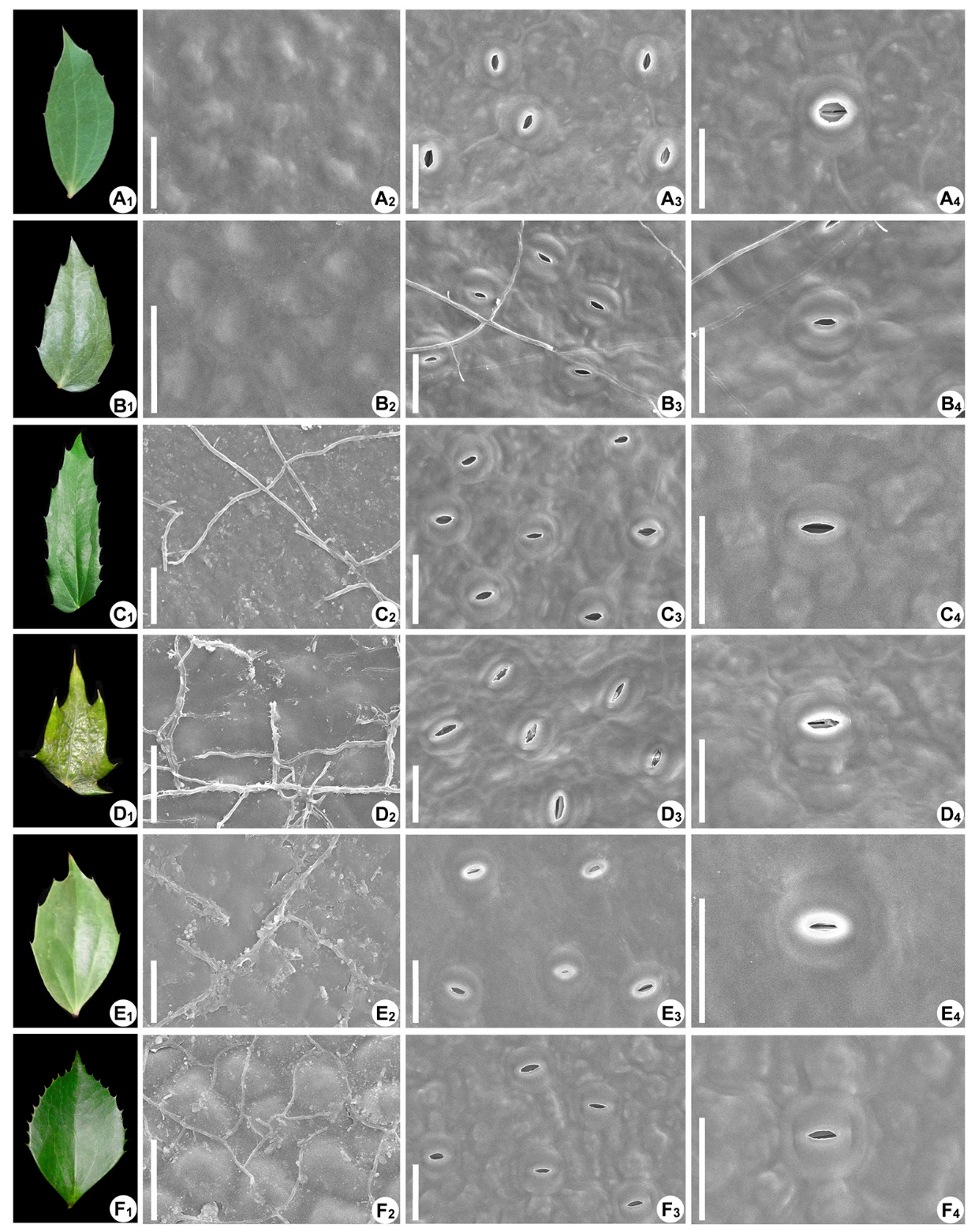


**Fig. S8** Characteristics of leaflets and epidermal surface.

**A_1_–A_4_** *M. shenii*. **B_1_–B_4_** *M. hancockiana*. **C_1_–C_4_** *M. duclouxiana*. **D_1_–D_4_** *M. cardiophylla*. **E_1_–E_4_** *M. nitens*. **F_1_–F_4_** *M. gracilipes.* The images show leaflets, adaxial leaves, abaxial leaves and magnifying stomatal apparatus on the abaxial surface in each row from the left to right, respectively.

**Table S1** Gene composition of the 20 complete *Mahonia* chloroplast genomes.

| Group of gene | Name of gene | | | | |
| --- | --- | --- | --- | --- | --- |
| Ribosomal RNA genes | *rrn16* (2) | *rrn23* (2) | *rrn4.5* (2) | *rrn5* (2) |  |
| Transfer RNA genes | *trnI-CAU* (2) | *trnI-GAU* (2) * | *trnL-UAA* * | *trnL-CAA* (2) | *trnL-UAG* |
|  | *trnR-UCU* | *trnR-ACG* (2) | *trnA-UGC* (2) * | *trnW-CCA* | *trnM-CAU* |
|  | *trnV-UAC* * | *trnV-GAC* (2) | *trnF-GAA* | *trnT-UGU* | *trnT-GGU* |
|  | *trnP-UGG* | *trnfM-CAU* | *trnG-UCC* | *trnG-GCC* * | *trnS-GGA* |
|  | *trnS-UGA* | *trnS-GCU* | *trnD-GUC* | *trnC-GCA* | *trnN-GUU* (2) |
|  | *trnE-UUC* | *trnY-GUA* | *trnQ-UUG* | *trnK-UUU* * | *trnH-GUG* |
| Small Subunit of ribosome | *rps2* | ***rps3* (2)** | *rps4* | *rps7* (2) | ***rps8* (2)** |
|  | ***rps11* (2)** | *rps12* (2) * | *rps14* | *rps15* | *rps16* |
|  | *rps18* | ***rps19* (2)** |  |  |  |
| Large Subunit of ribosome | *rpl2*(2) * | ***rpl14* (2)** | ***rpl16* (2) *** | *rpl20* | ***rpl22* (2)** |
|  | *rpl23* (2) | *rpl32* | *rpl33* | ***rpl36* (2)** |  |
| DNA-dependent RNA polymerase | *rpoB* | *rpoC1* * | *rpoC2* |  |  |
| Translational initiation factor | ***infA* (2)** |  |  |  |  |
| NADH dehydrogenase | *ndhA* * | *ndhB* (2) * | *ndhC* | *ndhD* | *ndhE* |
|  | *ndhF* | *ndhG* | *ndhH* | *ndhI* | *ndhJ* |
|  | *ndhK* |  |  |  |  |
| Subunits of photosystem I | *psaA* | *psaB* | *psaC* | *psaI* | *psaJ* |
|  | *ycf3* ** | *ycf4* |  |  |  |
| Subunits of photosystem II | *psbA* | ***psbB* (2)** | *psbC* | *psbD* | *psbE* |
|  | *psbF* | ***psbH* (2)** | *psbI* | *psbJ* | *psbK* |
|  | *psbL* | *psbM* | ***psbN* (2)** | ***psbT* (2)** | *psbZ* |
| Subunits of cytochrome | *petA* | ***petB* (2) *** | ***petD* (2) *** | *petG* | *petL* |
|  | *petN* |  |  |  |  |
| Subunits of ATP synthase | *atpA* | *atpB* | *atpE* | *atpF* * | *atpH* |
|  | *atpI* |  |  |  |  |
| Large subunit of Rubisco | *rbcL* |  |  |  |  |
| Maturase | *matK* |  |  |  |  |
| Membrane protein | *cemA* |  |  |  |  |
| Protease | *clpP* ** |  |  |  |  |
| Subunit of acetyl-CoA | *accD* * |  |  |  |  |
| C-type cytochrome synthesis gene | *ccsA* |  |  |  |  |
| Hypothetical reading frames | *ψycf1* | *ycf1* | *ycf2* (2) | *ycf15* (2) |  |

The number in braces mean genes with two copies, and single and double asterisks indicate genes with one and two introns, respectively. The 15 gene names in bold mean an additional copy due to the IRs expansion in *Mahonia*.

**Table S2** Numbers of nucleotide substitutions and pairwise sequence distance rate in *Mahonia* plastomes.

|  | *M.*  *bealei* RC601 | *M.*  *duclouxiana* RC602 | *M.*  *napaulensis*  RC603 | *M.*  *cardiophylla*  RC604 | *M.*  *nitens*  RC605 | *M.*  *gracilipes* RC606 | *M.*  *polyodonta*  RC607 | *M.*  *bodinieri*  RC608 | *M.*  *shenii*  RC609 | *M.*  *oiwakensis*  RC610 | *M.*  *breviracema*  RC611 | *M.*  *fordii*  RC612 | *M. hancockiana*  RC613 | *M. eurybracteata*  RC614 | *M. japonica*  RC615 | *M. aquifolium*  RC616 | *M. pinnata*  RC618 | *M.*  *bealei* MH795308 | *M. fortunei*  NC042167 | *M. ganpinensis*  MN417307 |
| --- | --- | --- | --- | --- | --- | --- | --- | --- | --- | --- | --- | --- | --- | --- | --- | --- | --- | --- | --- | --- |
| *M. bealei* RC601 |  | 0.00101 | 0.00096 | 0.00088 | 0.00194 | 0.00096 | 0.00338 | 0.00227 | 0.00086 | 0.00081 | 0.00074 | 0.00226 | 0.0012 | 0.00091 | 0.00189 | 0.00472 | 0.00526 | 0 | 0.00215 | 0.00094 |
| *M. duclouxiana*  RC602 | 167 |  | 0.00038 | 0.00091 | 0.00173 | 0.00065 | 0.00282 | 0.00197 | 0.00076 | 0.00055 | 0.00084 | 0.00199 | 0.00074 | 0.00085 | 0.00205 | 0.00425 | 0.00499 | 0.00102 | 0.00199 | 0.0009 |
| *M. napaulensis*  RC603 | 158 | 62 |  | 0.00077 | 0.00177 | 0.0007 | 0.00289 | 0.00208 | 0.00079 | 0.0005 | 0.00084 | 0.0021 | 0.00069 | 0.00092 | 0.00217 | 0.00437 | 0.00491 | 0.00098 | 0.00213 | 0.00079 |
| *M. cardiophylla*  RC604 | 145 | 150 | 127 |  | 0.00156 | 0.00064 | 0.00292 | 0.00208 | 0.00053 | 0.00074 | 0.00065 | 0.00214 | 0.00102 | 0.00071 | 0.00179 | 0.00433 | 0.00521 | 0.00088 | 0.00195 | 0.00074 |
| *M. nitens*  RC605 | 320 | 285 | 291 | 258 |  | 0.00164 | 0.00305 | 0.00087 | 0.00147 | 0.00171 | 0.00181 | 0.00092 | 0.00171 | 0.00164 | 0.00088 | 0.00458 | 0.00513 | 0.00192 | 0.00086 | 0.00161 |
| *M. gracilipes* RC606 | 158 | 108 | 115 | 106 | 271 |  | 0.00309 | 0.00186 | 0.0006 | 0.00064 | 0.00065 | 0.00191 | 0.00103 | 0.0007 | 0.00177 | 0.00441 | 0.00516 | 0.00099 | 0.00185 | 0.00069 |
| *M. polyodonta*  RC607 | 554 | 463 | 475 | 481 | 502 | 509 |  | 0.00305 | 0.00305 | 0.00277 | 0.00318 | 0.0032 | 0.00293 | 0.00301 | 0.00307 | 0.00426 | 0.00486 | 0.00336 | 0.00316 | 0.00308 |
| *M. bodinieri*  RC608 | 375 | 325 | 343 | 343 | 144 | 307 | 503 |  | 0.00171 | 0.00205 | 0.00199 | 0.00011 | 0.0017 | 0.00217 | 0.00018 | 0.00477 | 0.00536 | 0.00218 | 0.00099 | 0.00194 |
| *M. shenii*  RC609 | 143 | 126 | 130 | 88 | 242 | 99 | 502 | 283 |  | 0.00081 | 0.0007 | 0.00179 | 0.00107 | 0.00067 | 0.00161 | 0.00435 | 0.00511 | 0.00086 | 0.00173 | 0.00062 |
| *M. oiwakensis*  RC610 | 133 | 91 | 82 | 122 | 282 | 106 | 455 | 338 | 133 |  | 0.00078 | 0.00215 | 0.00075 | 0.00074 | 0.00217 | 0.00423 | 0.00498 | 0.00083 | 0.00209 | 0.00085 |
| *M. breviracema*  RC611 | 123 | 138 | 138 | 107 | 298 | 108 | 524 | 329 | 116 | 129 |  | 0.00207 | 0.00108 | 0.00084 | 0.00185 | 0.00449 | 0.00527 | 0.00074 | 0.00209 | 0.00077 |
| *M. fordii*  RC612 | 373 | 329 | 346 | 354 | 152 | 315 | 527 | 18 | 295 | 354 | 342 |  | 0.00166 | 0.00225 | 0.00022 | 0.00483 | 0.00539 | 0.00224 | 0.00098 | 0.00202 |
| *M. hancockiana*  RC613 | 199 | 123 | 113 | 169 | 283 | 170 | 482 | 281 | 177 | 124 | 179 | 275 |  | 0.00121 | 0.00183 | 0.00429 | 0.00504 | 0.00123 | 0.00192 | 0.00106 |
| *M. eurybracteata*  RC614 | 150 | 141 | 152 | 117 | 271 | 116 | 496 | 359 | 111 | 123 | 139 | 372 | 200 |  | 0.00186 | 0.00436 | 0.00498 | 0.00091 | 0.00189 | 0.0004 |
| *M. japonica*  RC615 | 313 | 339 | 358 | 296 | 145 | 292 | 506 | 30 | 266 | 358 | 305 | 36 | 303 | 307 |  | 0.00488 | 0.00546 | 0.00199 | 0.00107 | 0.00162 |
| *M. aquifolium*  RC616 | 777 | 700 | 718 | 714 | 755 | 727 | 701 | 786 | 717 | 697 | 740 | 795 | 707 | 719 | 804 |  | 0.00113 | 0.00472 | 0.00494 | 0.00431 |
| *M. pinnata*  RC618 | 868 | 823 | 809 | 860 | 845 | 851 | 800 | 884 | 842 | 821 | 869 | 888 | 830 | 821 | 900 | 186 |  | 0.00527 | 0.00542 | 0.00511 |
| *M. bealei* MH795308 | 0 | 169 | 162 | 145 | 318 | 163 | 553 | 361 | 143 | 137 | 123 | 371 | 203 | 151 | 329 | 778 | 869 |  | 0.00215 | 0.00096 |
| *M. fortunei*  NC042167 | 356 | 329 | 351 | 322 | 142 | 305 | 521 | 164 | 285 | 344 | 345 | 162 | 318 | 313 | 177 | 814 | 894 | 355 |  | 0.00181 |
| *M. ganpinensis* MN417307 | 155 | 149 | 131 | 123 | 266 | 114 | 507 | 320 | 102 | 140 | 128 | 333 | 175 | 66 | 267 | 710 | 843 | 159 | 199 |  |

The upper triangle shows the number of nucleotide substitutions and the lower triangle indicates the number of sequence distance in the *Mahonia* plastomes.

**Table S3** Akaike information criterion (AICc) selection results for nine datasets.

|  | **Plastome** | **LSC** | **SSC** | **IR** | **CDS** | **Non-CDS** | **ITS** | **Barcode** | **Hotspots** |
| --- | --- | --- | --- | --- | --- | --- | --- | --- | --- |
| **Model** | GTR+I+G | GTR+I+G | GTR+I+G | TVM+I | TVM+I+G | GTR+I+G | GTR+I | TIM1+I+G | TVM+I+G |
| **partition** | 12345 | 12345 | 12345 | 12314 | 12314 | 12345 | 12345 | 12230 | 12314 |
| **-lnL** | 445488.0398 | 231009.7842 | 64732.6036 | 50883.8814 | 198782.3052 | 272161.4470 | 3123.3996 | 10642.3894 | 55348.2798 |
| **K** | 68 | 68 | 68 | 66 | 67 | 68 | 63 | 66 | 67 |
| **freqA** | 0.3157 | 0.3288 | 0.3413 | 0.2945 | 0.3086 | 0.3241 | 0.2128 | 0.3509 | 0.3239 |
| **freqC** | 0.1845 | 0.1752 | 0.1642 | 0.2015 | 0.1776 | 0.1764 | 0.3016 | 0.1614 | 0.1569 |
| **freqG** | 0.1799 | 0.1700 | 0.1464 | 0.2155 | 0.2046 | 0.1771 | 0.2978 | 0.1834 | 0.1581 |
| **freqT** | 0.3199 | 0.3260 | 0.3482 | 0.2886 | 0.3092 | 0.3225 | 0.1877 | 0.3043 | 0.3612 |
| **ti/tv** | - | - | - | - | - | - | - | - | - |
| **R(a)** | 0.9285 | 0.9483 | 0.7393 | 1.1725 | 1.5758 | 0.9957 | 1.1151 | 1.0000 | 0.9588 |
| **R(b)** | 2.0008 | 2.1156 | 1.6024 | 2.1369 | 3.1532 | 1.9220 | 2.4996 | 1.4831 | 1.8421 |
| **R(c)** | 0.6451 | 0.5908 | 0.4509 | 0.6345 | 0.5738 | 0.8171 | 1.2916 | 0.6001 | 0.4917 |
| **R(d)** | 0.4586 | 0.5056 | 0.4872 | 0.5030 | 0.8114 | 0.4336 | 0.4273 | 0.6001 | 0.9103 |
| **R(e)** | 1.9407 | 1.9170 | 1.7357 | 2.1369 | 3.1532 | 1.8074 | 4.0644 | 1.8865 | 1.8421 |
| **R(f)** | 1.0000 | 1.0000 | 1.0000 | 1.0000 | 1.0000 | 1.0000 | 1.0000 | 1.0000 | 1.0000 |
| **p-inv** | 0.4700 | 0.3140 | 0.2440 | 0.7630 | 0.4280 | 0.3690 | 0.3430 | 0.4110 | 0.0970 |
| **gamma** | 0.9660 | 0.9200 | 0.9350 | - | 0.9180 | 0.9030 | - | 0.7720 | 0.7750 |
